# Road safety measurement with reliability using an advanced hybrid decision model

**DOI:** 10.1038/s41598-025-18918-7

**Published:** 2025-10-08

**Authors:** Jiaxu Jin, Hanrui Feng, Haojing Gao, Hongyang Hua, Mingshuo Liu, Hedong Liang, Hongping Qi, Lei Sun, Fang Tian, Jiachen Yao, Aaron Kaiqiang Zhou, Yiyun Zhang, Xingjian Zhang, Ziyan Li, Dongxu Qin, Mingren Zheng, Haocheng Yang, Faan Chen

**Affiliations:** 1 Financial Market Department, Zhongyuan Bank Co., Ltd, Zhengzhou, 450000 Henan China; 2 Department of Mathematics, University of Chicago, Chicago, IL 60637 USA; 3 Grainger College of Engineering, University of Illinois Urbana-Champaign, Urbana, IL 61801 USA; 4 Talent Program from China Association for Science and Technology, Beijing Science Center, Ministry of Education, Beijing, 100190 China; 5 Department of Software Engineering, Northeastern University, Shenyang, 110169 Liaoning China; 6 School of Mechatronic Engineering, Xi’an Institute of Electromechanical Information Technology, Xi’an, 710065 Shaanxi China; 7 College of Letters and Science, University of California at Davis, Davis, CA 95616 USA; 8 School of Engineering and Applied Science, University of Pennsylvania, Philadelphia, PA 19104 USA; 9 School of Architecture, The Chinese University of Hong Kong, Hong Kong, Hong Kong SAR China; 10 College of Environmental Science and Engineering, Tongji University, Shanghai, 200092 China; 11 Schulich School of Business, York University, Toronto, ON M3J 1P3 Canada; 12 School of Social Sciences, University of California at San Diego, San Diego, CA 92093 USA; 13 Courant Institute of Mathematical Sciences, New York University, New York, NY 10012 USA; 14 College of Radio and Television, Communication University of China Nanjing, Nanjing, 211172 Jiangsu China; 15 Department of Philosophy, Syracuse University, Syracuse, NY 13210 USA; 16 Tabor Academy, Marion, MA 02738 USA; 17 School of Information Science and Engineering, Nanjing University Jinling College, Nanjing, 210031 Jiangsu China; 18 School of Engineering and Applied Sciences, Harvard University, Cambridge, MA 02138 USA

**Keywords:** Multi-criteria decision-making (MCDM), Decision reliability, Road safety, Policy making, East asia summit (EAS), Engineering, Civil engineering

## Abstract

This study proposes a brand-new hybrid multi-criteria decision-making (MCDM) framework that combines High-Dimensional Vector Projection (HDVP) and Between-class Variance Maximization (BeVarMax), termed the HDVP–BeVarMax model, aiming to provide trustworthy decisions and defensible policy conclusions. Specifically, HDVP quantifies the relative proximity of each country to an ideal performance vector in a high-dimensional space, ensuring scale-invariant and geometrically meaningful aggregation. BeVarMax, inspired by Otsu’s thresholding method, maximizes between-class variance to identify optimal groupings and uncover latent structure among alternatives, surpassing conventional clustering techniques such as k-means in robustness and global optimality. Using longitudinal data from 13 East Asia Summit (EAS) countries spanning 2012 to 2023, this model is applied to measure national road safety performance based on 15 tailored safety performance indicators (SPIs). Results demonstrate the model’s reliability, robustness, and superior discriminative power across normalization and weighting schemes, validated through extensive sensitivity and benchmarking analyses. Policy implications are twofold: it enables benchmarking of high and low performers to guide targeted interventions, and it supports strategic resource allocation by identifying priority areas such as enforcement, infrastructure, and behavioral factors. The proposed model serves as a practical decision-support tool for monitoring progress and fostering regional cooperation in line with global road safety goals.

**Supplementary Information:**

The online version contains supplementary material available at 10.1038/s41598-025-18918-7.

## Introduction

**Fig. 1:**
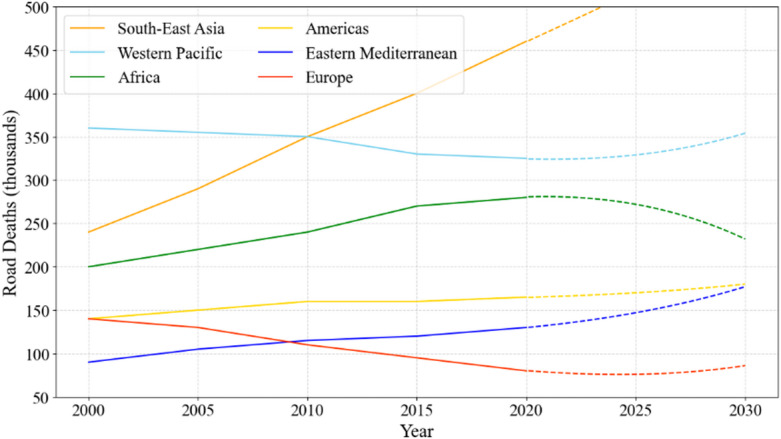
Trends of road deaths across the world.

Road safety engineering is a critical factor in the infrastructure and development strategy of countries all over the world, contributing significantly to the safety and well-being of citizens as well as to economic growth. Globally, road accidents result in about 1.35 million fatalities each year, a figure that stands starkly against the backdrop of 55 million deaths from all causes annually^1^. This highlights a significant shortfall in road safety measures across the world and underscores the critical need for improvements to prevent such distressing losses. This is especially true for the East Asia Summit (EAS) nations, which jointly account for over 50% of the global population and over 60% of the world’s yearly gross domestic product (GDP)^2^, and have been encountering substantial challenges in road safety, as shown in Fig. 1. Though concerted efforts have been made over the past decade to enhance road safety standards, which recognize the direct impact of such measures on national development, there is still a conspicuous absence of a uniform and standardized framework for measuring and monitoring road safety across the EAS region, which involves multiple criteria and alternatives. Therefore, a framework that can not only report road safety performance but also serve as a catalyst for policy formulation and implementation is desperately required.

Deriving effective decision outcomes and formulating sound policy recommendations involves various safety performance indicators (SPIs) and alternatives (countries) evaluated, which largely rely on the principles of multi-criteria decision-making (MCDM). This necessitates the integration of various analytical methods (e.g., aggregating, grouping) into a cohesive evaluation framework. MCDM model functions as a vital support mechanism in decision processes, especially when navigating complex problems characterized by numerous objectives or evaluation criteria. It assists decision-makers in selecting the most appropriate alternative from a set of competing options by systematically handling trade-offs and establishing priorities among the various factors involved.

Previous studies have established a solid methodological foundation in this regard. Although notable progress has been made, several research gaps persist, providing key motivations for the present study: (1) The absence of a commonly accepted framework for SPIs at a regional scale within the EAS limits the applicability and impact of current evaluation models. (2) Many existing approaches fall short in effectively managing the classification, deconstruction, and de-composition of alternatives; processes that are essential for extracting actionable insights and are critical to informed decision-making. Integrated frameworks that simultaneously address the processes of aggregation, classification, deconstruction, and de-composition remain scarce. (3) The influence of normalization manners, weighting schemes, aggregation techniques, and clustering operations on the propagation of model uncertainty has received limited attention, thereby weakening the consistency and robustness of decisions, especially when applied to datasets of limited or moderate size. (4) Prior studies have predominantly concentrated on single-nation contexts or smaller subregional applications, seldom extending to cross-border scenarios that involve countries with differing socio-economic structures, such as EAS countries. Consequently, it is essential to design a structured and empirically validated MCDM framework capable of addressing diverse geographical settings. This framework should encompass all key components of the decision-making process, such as index aggregation, alternative categorization, analytical breakdown, and detailed de-composition, while maintaining a strong emphasis on achieving operational efficiency, decision consistency, and model dependability.

To this end, this study proposes a brand-new MCDM methodology that integrates the High-Dimensional Vector Projection (HDVP) and Between-class Variance Maximization (BeVarMax), termed HDVP–BeVarMax model. Multifaceted risks and uncertainties associated with road transportation, such as vehicle-related risks and enforcement uncertainty, create a complex, high-dimensional decision environment where traditional single-criterion or rigid models often fall short. The proposed HDVP–BeVarMax framework is designed to accommodate this complexity by integrating multi-indicator performance data, enabling both aggregation and classification that reflect the nuanced realities of cross-national road safety systems, and aiming to offer a reliable decision-making and policy formulation tool concerning road safety development for the EAS countries.

This study contains original value and several contributions: (1) A tailored SPIs framework is introduced, offering a holistic basis for evaluating, tracking, and benchmarking road safety performance across the EAS region. (2) The study enhances methodological advancement by proposing a brand-new MCDM framework (i.e., HDVP–BeVarMax model), enriching the MCDM methodological database. This approach integrates various stages of the MCDM process, i.e., aggregating, grouping, and deconstructing, into a single streamlined procedure. (3) Compared to traditional MCDM methods such as TOPSIS and k-means, the HDVP–BeVarMax model offers several methodological and practical enhancements. The HDVP component of the proposed model retains the geometric integrity of high-dimensional data, offering a more robust and scale-invariant evaluation of each alternative’s proximity to the ideal vector. Simultaneously, BeVarMax overcomes the clustering limitations of methods like k-means by maximizing between-class variance, ensuring globally optimal grouping outcomes and better uncovering structural patterns in national performance profiles. This dual-framework not only enhances ranking stability and cluster interpretability but also strengthens the policy utility of the model by clearly distinguishing top performers, identifying performance bottlenecks, and enabling peer learning. (4) Practical recommendations and strategic insights are delivered for EAS countries, supporting the development of policies aimed at minimizing traffic-related incidents, lowering the rates of injuries and fatalities, alleviating economic burdens, and contributing to a safer transportation system. These efforts are in alignment with the Sustainable Development Goal to halve global road traffic deaths by the year 2030.

The structure of this paper is organized as follows. Section Literature review reviews existing approaches in regard to aggregating and grouping. Section Data introduces the SPIs system applied in this study and related data sources. Section Methodology presents the details of the full proposed methodology. Section Results and discussion reports and discusses the empirical results. Section Policy and practical guidance provides a series of practical guidelines for future road safety management, followed by the concluding remarks in Sect. Concluding remarks.

## Literature review

MCDM has emerged as one of the primary decision methodologies over the past decades, finding wide-ranging applications across numerous fields. While the initial MCDM methods have proven instrumental in many applications, it is noteworthy that they may not necessarily be appropriate for every problem or scenario. As a result, numerous improved versions and adaptations of these methods have emerged over time. These refined techniques have not only broadened the applicability of MCDM but have also enhanced its versatility, precision, and reliability. For instance, hybrid MCDM methods integrating two or more of these techniques have been developed, providing better decision-making support in more complex and multifaceted scenarios^3,4^. The evolution of these methods has also been facilitated by advances in computational and analytical capabilities, allowing for more robust, rigorous, and complex decision-making processes.

### MCDM methods

#### Aggregating methods

Aggregating is the act of combining separate indicators into a unified index using a foundational model. Road safety analyses rely heavily on the systematic application of aggregation methods and integral processes in the creation of composite indices. Developed over time, these methods chiefly fall into two categories: linear and geometric aggregation^5,6^. In the realm of linear aggregation, the weighted sum method stands as a prevalent technique, widely adopted for index aggregation due to its practicability. Conversely, the geometric aggregation field is primarily dominated by the MCDM approaches. Classical methods used for aggregating MCDM operations see Appendix A.

These methods have been found to have extensive application across various sectors, including road safety. They provide a robust framework for risk factor assessment, safety measure evaluation, and intervention prioritization. However, its successful application depends on the specific context and demands careful consideration of various factors, including the road network characteristics, data availability, involved stakeholders, and the policy context. Despite the inherent complexities and potential limitations, the evolution of aggregation methods, particularly MCDM, underscores their pivotal role in advancing road safety.

#### Grouping methods

Grouping is the process of organizing and classifying related samples or products into meaningful, coherent groups based on similarities or patterns among the data points. In the field of road safety, the group method plays a significant role, providing a means to assess, compare, and improve performance across different countries or regions^7^. Frequently used grouping methods see Appendix A.

These methods provide a systematic approach to road safety analysis, enabling comparison of different countries or regions within their respective groups. By identifying the best performers in each group, these techniques facilitate learning from the ‘best in class’, fostering the improvement of road safety measures and strategies globally.

#### Hybrid MCDM methods

Many studies have applied MCDM methods across diverse fields such as business, engineering, environment, healthcare, and public policy^8^. However, no single MCDM method is universally optimal for every decision problem^9^.

To address the increasing complexity and multidimensionality of decision problems, researchers have developed hybrid MCDM methods as a promising solution. Decision-making models are made more robust and applicable by integrating multiple methodological frameworks. According to Zavadskas, hybrid MCDM methods usually combine four main categories of decision-making techniques or their combinations. These include methods for calculating criteria weights, as well as tools such as fuzzy sets and gray systems for managing uncertainty^10^.

Typically, multi-criteria ranking techniques (e.g., TOPSIS, VIKOR) are paired with weighting methods such as SAW, Entropy, CRITIC, or AHP^11,12^. This approach has been applied across various domains. Liu et al.^11^ proposed an improved entropy-weighted TOPSIS method for evaluating decision-level fusion in multi-source data systems, and in the energy sector, Arslan et al.^13^ applied an AHP–TOPSIS hybrid model to evaluate geothermal energy system designs. Similarly, Youssef and Saleem^14^ developed a hybrid MCDM model combining BWM, SAW, and Delphi methods to evaluate web-based e-learning platforms. Besides, Sadhu et al.^15^ applied ANN-AHP to evaluate the overall acceptance of the optimized conditions.

Furthermore, approaches such as fuzzy logic and gray systems, which are used to model uncertainty, have been combined with MCDM methods to handle uncertainty in decision-making^16^. Cao and Xu^17^ proposed an entropy-based fuzzy TOPSIS framework to support the optimization of investment decisions in large-scale projects. In banking applications, Chaurasiya and Jain^18^ developed a hybrid Pythagorean fuzzy MCDM model integrating PF-MEREC, SWARA, and COPRAS to evaluate banking management systems. In civil engineering, Nila et al.^19^ applied F-CoCoSo to evaluate and find the most suitable drone-based city logistics concept. Furthermore, Sharma et al.^20^ employed LOPCOW-DOBI to evaluate and rank multiple suppliers based on normal business criteria and resilient pillars.

### MCDM applications in road safety measurement

Road safety assessment requires evaluating multiple factors, including infrastructure conditions, traffic patterns, driver behavior, and environmental conditions. Single-criterion evaluation methods often cannot handle this complexity effectively. This creates challenges for decision-makers. They need to prioritize safety improvements with limited budgets^21^. MCDM methods provide systematic approaches for handling these multi-factor problems. These methods have been applied to various aspects of road safety, including identifying dangerous locations, evaluating infrastructure improvements, and comparing safety policies. Several studies have applied MCDM methods to identify and rank hazardous locations in road networks. For instance, Fancello et al.^21^ applied MCDM techniques including TOPSIS, ELECTRE III, and VIKOR to rank hazardous intersections within urban networks. This approach enabled more effective allocation of limited safety resources. Similarly, Stević et al.^22^ proposed a hybrid model integrating IMF-SWARA and EDAS to assess road sections using factors such as AADT and accident severity. Fancello et al.^23^ employed two DEA models to rank road segments. They used traffic flow and conflict points as inputs and the social cost of accidents as the sole output. These studies show that MCDM frameworks support better decision-making for road safety in urban networks. Building on similar approaches, Vrtagić et al.^24^ developed an integrated fuzzy model combining improved fuzzy SWARA (IMF SWARA) with fuzzy MARCOS for ranking road sections based on safety degrees.

Beyond hazardous location identification, MCDM approaches have been applied in various other domains of road safety. For instance, Wang et al.^25^ developed an entropy–CoCoSo-based MCDM framework to objectively rank OECD countries based on road transport sustainability indicators. Recent developments have also integrated MCDM methods with machine learning techniques in transport safety studies. Zhou et al.^26^ proposed a hybrid model combining MEREC, CoCoSo, and DBSCAN for transport safety planning in OAS countries. The model incorporates UMAP and KNN algorithms to automate parameter selection. Guo et al.^27^ developed a model integrating LOPCOW, MULTIMOORA, and DBSCAN with grid search optimization.

MCDM methods have been applied to infrastructure safety assessment and risk evaluation. Trivedi et al.^28^ developed a hybrid BWM-TOPSIS-SAW approach to prioritise road safety improvements across different sections of the road. Various criteria were analyzed in the study to provide a systematic evaluation of safety improvements. Ghoushchi et al.^29^ proposed an integrated SWARA-MARCOS approach in a spherical fuzzy environment for assessing road safety risks. They applied this methodology to rural roads. They used failure mode and effect analysis (FMEA). The results showed human factors as the most significant risk source compared to environmental factors. Farooq et al.^30^ used an integrated AHP-BWM model to evaluate the factors influencing frequent lane-changing behavior. These studies show that MCDM is effective in dealing with a variety of road safety issues.

### Research gap

Despite notable progress in road safety evaluation, existing studies remain constrained by several critical gaps: the absence of a commonly accepted regional framework for safety performance indicators (SPIs), limited attention to uncertainty propagation across normalization, weighting, and clustering stages, and the insufficient adaptability of current models to multi-country, heterogeneous datasets. Moreover, most prior applications rely on either aggregation or clustering in isolation, which restricts their ability to simultaneously deliver robust rankings and interpretable group structures.

Addressing these gaps, this study introduces the HDVP–BeVarMax model, a novel hybrid framework that integrates HDVP for scale-invariant aggregation with BeVarMax for robust grouping. This dual mechanism not only enhances ranking stability and cluster interpretability but also strengthens the methodological link between evaluation and classification, ensuring both rigor and policy relevance. By combining aggregation, classification, and de-composition into a unified model, the study advances current knowledge by offering a more systematic, reliable, and practically applicable decision-support tool for benchmarking road safety performance at a regional scale.

## Data

### SPIs

**Fig. 2:**
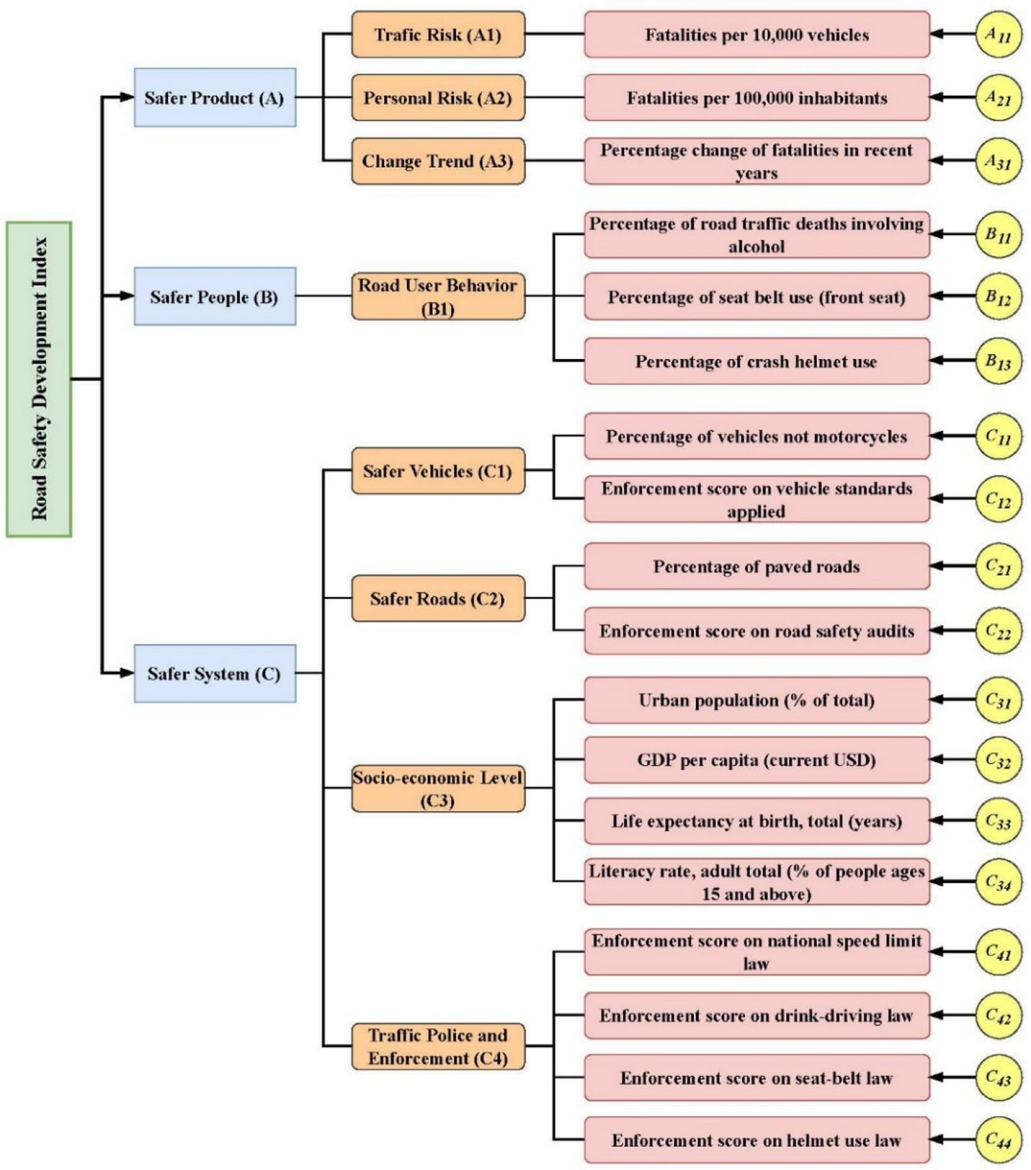
SPIs system adopted in this study.

Over the past decades, numerous studies have proposed a wide range of indicators designed to evaluate and track the status of road safety, including road safety index^31^, road safety performance index^32^, hierarchically structured safety performance indicators^33^, road safety performance index for crash prediction^34^, optimized success indicator^35^, and optimal road safety composite index^36^, forming a foundational basis for the present study. Based on the literature review, a set of SPIs comprising 15 indicators are determined and generated, as presented in Fig. 2.

### Data collection

Dataset on the SPIs is collected for 13 EAS countries. Specifically, indicators related to fatalities and motorcycle use, namely A11, A21, and C11, were derived using statistics on road traffic deaths, vehicle registration, and population figures sourced from ASEANStats^37^ and multiple editions of the World Health Organization (WHO) global status report^1,38–41^. Information reflecting road user behavior and enforcement practices, specifically indicators B11, B12, B13, C41, C42, C43, and C44, was obtained directly from WHO global reports^1,38–41^. Data concerning road infrastructure (C21) were compiled from various authoritative sources, including ASEANStats ^37^, the ASEAN-Japan Transport Partnership data center^42^, the United Nations Economic and Social Commission for Asia and the Pacific (UN ESCAP)^43^, the CIA World Factbook^44^, and the International Road Federation^45^. Socio-economic indicators (C31, C32, C33, and C34) were sourced from the World Bank’s international databases^46–49^. Additionally, enforcement-related variables (C12 and C22) were extracted based on data provided in the WHO global reports^1,38–41^.

## Methodology

In the context of MCDM, systematic and robust are two important qualities of the model. In this study, a model is defined as systematic if it integrates multiple interdependent stages of the decision-making process, such as data normalization, indicator weighting, multidimensional aggregation, grouping, and performance de-composition, within a coherent and unified analytical framework. Robustness refers to the degree to which the model’s outputs, such as rankings, scores, and groupings, remain stable and consistent under varying methodological conditions. These conditions include different normalization techniques, weighting schemes, and alternative benchmark models.

### Proposed methods

In this study, we propose a systematic hybrid model named HDVP–BeVarMax that incorporates the proposed HDVP model for data aggregating and a novel BeVarMax approach for clustering. The motivation behind developing this model is threefold: (1) To address the limitations of traditional MCDM approaches, which often struggle with scale invariance and interpretability when applied to multi-country data with heterogeneous indicators; (2) To provide a geometrically intuitive and computationally robust aggregation technique (HDVP) that maintains the integrity of high-dimensional performance space; and (3) To develop a global-optimum, variance-maximizing clustering mechanism (BeVarMax) that avoids the randomness and local minima issues inherent in widely used clustering algorithms like k-means.

We develop HDVP as an aggregating method for data measurement over road safety. HDVP describes the performance of all the alternatives in the high-dimensional space, and scores the overall performance for each alternative by smartly projecting its performance vector onto the base vector that is defined as the vector pointing from the worst possible performance point towards the best possible performance point in this high-dimensional space. Though both HDVP and TOPSIS^50^ quantify the performance in the high-dimensional space, HDVP has stronger geometric significance and higher intuitivity than TOPSIS, which directly and fairly measures how well an alternative performs from the perspective of the high-dimensional space. HDVP operates by projecting each alternative’s performance vector onto the base vector in a high-dimensional space formed between the worst and best possible performance points. This approach ensures scale invariance, retains the directionality and shape of the data distribution, and avoids the need for distance normalization between ideal and anti-ideal points. In contrast, TOPSIS computes Euclidean distances to ideal/anti-ideal solutions, which can distort proportional relationships among indicators, especially when the data are highly asymmetric or skewed. The side-by-side comparison outlining HDVP and TOPSIS across key criteria (i.e., interpretability, complexity, sensitivity, and scalability) is presented in Table 1.

**Table 1:** Side-by-side comparison between HDVP and TOPSIS.

Criterion	HDVP	TOPSIS
Interpretability	High – Scores are derived from vector projection, making the geometric logic transparent and intuitive.	Moderate – Based on relative Euclidean distances, which may be less intuitive to non-experts.
Complexity	O(n), where *n* is the number of criteria – vector projection and normalization.	O(n) – requires computing two Euclidean distances per alternative.
Sensitivity	Low – Projection-based scoring is scale-invariant, reducing distortion from outliers.	High – Sensitive to normalization method, especially under skewed or uneven data.
Scalability	High – Performs efficiently with large numbers of alternatives and criteria.	High – Also scales well computationally, but results may be more affected by dimensionality.

On the basis of HDVP, we further develop a new grouping method named BeVarMax, which is partially inspired by Otsu’s thresholding method^51^ in the realm of image processing. BeVarMax is particularly well-suited for this study because it explicitly maximizes between-class variance, making it ideal for uncovering structural group differences in cross-national performance profiles, especially when the data do not conform to spherical or uniformly distributed clusters, as is often the case in multi-country policy datasets. Unlike k-means, which minimizes intra-cluster distances and is sensitive to initial centroid selection and data scaling, BeVarMax deterministically finds globally optimal thresholds based on histogram distribution, thereby offering greater stability, interpretability, and reproducibility. This is crucial when policy implications are drawn from the resulting group structures. The trade-offs involve computational efficiency and flexibility. BeVarMax, due to its exhaustive search for optimal thresholds, has higher computational complexity than k-means or DBSCAN, especially as the number of clusters increases. Moreover, BeVarMax is currently limited to unidimensional clustering based on the aggregated performance scores, while methods like DBSCAN operate in multidimensional space and can detect arbitrary-shaped clusters. However, given our goal of producing transparent, policy-relevant groupings from interpretable composite indices, we find that BeVarMax provides the most appropriate balance of statistical rigor and practical utility in this context.

The integration of HDVP and BeVarMax within a unified MCDM framework offers notable methodological and practical advantages in evaluating and categorizing road safety performance. HDVP allows for an intuitive and geometrically meaningful aggregation of multi-criteria data by measuring the closeness of each alternative to an ideal performance vector. This approach ensures that the evaluation is both scale-invariant and sensitive to the relative positioning of alternatives in a multidimensional performance space. However, while HDVP effectively provides a composite score for ranking, it does not inherently address the need to group countries with similar performance characteristics. By coupling HDVP with BeVarMax, the model enhances its capability to reveal structural patterns in the data. The combined framework not only strengthens decision support by linking evaluation and classification into a seamless process but also improves the interpretability of results for benchmarking and policy learning. This synergy allows for a more nuanced understanding of both absolute performance and relative positioning, enabling policymakers to identify not only who is performing well, but also which countries exhibit similar profiles, thereby facilitating peer-to-peer learning and targeted interventions.

### Model specification: HDVP–BeVarMax model

**Step 1: Decision matrix building.**

Given an MCDM problem having *m* alternatives, each alternative includes *n* criteria, and we use *x*_*ij*_ to represent the performance value of alternative *i* (*i* = 1, 2, *…*,* m*) criterion *c*_*j*_ (*j* = 1, 2, *. .*,* n*). The original matrix can be written as:

1 $$X=\left[ {\begin{array}{*{20}{c}} {{x_{11}}}&{{x_{12}}}& \cdots &{{x_{1n}}} \\ {{x_{21}}}&{{x_{22}}}& \cdots &{{x_{2n}}} \\ \vdots & \vdots & \ddots & \vdots \\ {{x_{m1}}}&{{x_{m2}}}& \cdots &{{x_{mn}}} \end{array}} \right]$$

**Step 2. Direction transformation of each indicator**.

For benefit (positive) criteria (*A*_11_, *A*_21_, *A*_31_ and *B*_11_ in this study):

2 $${y_{ij}}={x_{ij}}$$

For cost (negative) criteria (the remaining indicators in this study):

3 $${y_{ij}}=\frac{1}{{{x_{ij}}}}$$

**Step 3: Vector normalization over the data.**

Since the decision matrix *Y* has not been normalized, making the scale of the criteria quite different, the next step lies in normalizing the decision matrix by the vector normalization method^52^.

4 $${z_{ij}}=\frac{{{y_{ij}}}}{{\sqrt {\sum\limits_{{k=1}}^{m} {{{({y_{kj}})}^2}} } }}$$

Hence, the normalized decision matrix can be denoted as:

5 $$Z=\left[ {\begin{array}{*{20}{c}} {{z_{11}}}&{{z_{12}}}& \cdots &{{z_{1n}}} \\ {{z_{21}}}&{{z_{22}}}& \cdots &{{z_{2n}}} \\ \vdots & \vdots & \ddots & \vdots \\ {{z_{m1}}}&{{z_{m2}}}& \cdots &{{z_{mn}}} \end{array}} \right]$$

**Step 4: Indicator weighting using DCRITIC.**

The weight *w*_*j*_ of indicator *j* can be computed by DCRITIC^53^ as follows:

6 $$dCor({c_j},{c_{j^{\prime}}})=\frac{{dCov({c_j},{c_{j^{\prime}}})}}{{\sqrt {dVar({c_j})dVar({c_{j^{\prime}}})} }}$$

where *d* Cov (*c*_*j*_, *c*_*j*’_) is the distance covariance between criteria *c*_*j*_ and *c*_*j*’_, *d* Var (*c*_*j*_) = *d* Cov (*c*_*j*_, *c*_*j*_) is the distance variance of *c*_*j*_, and *d* Var (*c*_*j*_’) = *d* Cov (*c*_*j*’_, *c*_*j*’_) is the distance variance of *c*_*j*’_.

The amount of information contained in criterion *j* is calculated by applying:

7 $${I_j}={s_j}\sum\limits_{{j'=1}}^{n} {\left( {1 - dCor({c_j},{c_{j'}})} \right)}$$

where *s*_*j*_ is calculated as:

8 $${s_j}=\sqrt {\frac{{{{\left( {\sum\limits_{{i=1}}^{m} {{z_{ij}} - {{\bar {z}}_j}} } \right)}^2}}}{{m - 1}}}$$

where ${\bar {z}_j}$ is the mean score of criterion *j*, and *m* is the total number of alternatives.

Then, the final weight of criterion *j* can be computed as:

9 $${w_j}=\frac{{{I_j}}}{{\sum\limits_{{j=1}}^{n} {{I_j}} }}$$

**Step 5: Computing the composite scores for alternatives using HDVP.**

In this study, a novel multi-criteria aggregation method named HDVP is proposed and employed to calculate the measurement scores of the alternatives.

The core idea of HDVP is to describe the performance of the alternatives in the high-dimensional space. The very first step is to find the best and worst performance points across all the criteria in the high-dimensional space. Specifically, for criterion *j*, we can compute the best-performing alternative as $z_{j}^{{\hbox{max} }}=\hbox{max} \{ {z_{1j}},{z_{2j}}, \cdots ,{z_{mj}}\}$and the worst-performing alternative as $z_{j}^{{\hbox{min} }}=\hbox{min} \{ {z_{1j}},{z_{2j}}, \cdots ,{z_{mj}}\}$, so that the best-performing point possible in the high-dimensional space is represented as: ${z^{\hbox{max} }}={[z_{1}^{{\hbox{max} }},z_{2}^{{\hbox{max} }}, \cdots ,z_{n}^{{\hbox{max} }}]^T} \in {{\mathbb{R}}^n}$, and similarly, the worst-performing point is denoted as: ${z^{\hbox{min} }}={[z_{1}^{{\hbox{min} }},z_{2}^{{\hbox{min} }}, \cdots ,z_{n}^{{\hbox{min} }}]^T} \in {{\mathbb{R}}^n}$.

Now if we consider the worst-performing point ${z^{\hbox{min} }}$ as the origin and the difference between best-performing point and the worst-performing point: ${v^*}=({z^{\hbox{max} }} - {z^{\hbox{min} }})$ as the vector where each entry is the upper-bound value for each criterion, then an alternative’s performance can be geometrically described by a vector pointing from this origin to its high-dimensional point: ${z_i}={[{z_{i1}},{z_{i2}}, \cdots ,{z_{in}}]^T} \in {{\mathbb{R}}^n}$. Note that point ${z_i}$ must lie inside the high-dimensional ‘box’ formed by ${z^{\hbox{min} }}$ and ${z^{\hbox{max} }}$ as two opposite corner points. Thus, this performance vector of alternative *i* can be denoted as: ${v_i}=({z_i} - {z^{\hbox{min} }})$.

In this manner, if we project the performance vector of an alternative *v*_*i*_ onto vector v^*^, we can obtain a scalar that quantifies how close it is between *v*_*i*_ and v^*^. Thus, by normalizing this scalar by the length of v^*^, we can achieve our scale-invariant aggregation score for alternative *i* such that: ${E_i}=\frac{{v_{i}^{T}{v^*}}}{{||{v^*}||}}$.

**Step 5.1: Determination of ideal performance boundaries**.

In the m-dimensional performance space, we first identify the optimal and worst values for each criterion j, determined as follows:

10 $$\begin{gathered} v_{j}^{+}={\hbox{max} _i}\left\{ {{x_{ij}}} \right\} \hfill \\ v_{j}^{ - }={\hbox{min} _i}\left\{ {{x_{ij}}} \right\} \hfill \\ \end{gathered}$$

This step establishes the best-performing vector $\:{V}^{+}=\left({v}_{1}^{+},{v}_{2}^{+},\dots\:,{v}_{n}^{+}\right)$ and worst-performing vector $\:{V}^{-}=\left({v}_{1}^{-},{v}_{2}^{-},\dots\:,{v}_{n}^{-}\right)$.

**Step 5.2: Construction of the base vector**.

Next, a base vector $\:{v}^{*}$ is defined to represent the direction of improvement from the worst-performing point toward the ideal performance point:

11 $${v^*}={V^+} - {V^ - }=(v_{1}^{+} - v_{1}^{ - },v_{2}^{+} - v_{2}^{ - }, \cdots ,v_{n}^{+} - v_{n}^{ - })$$

**Step 5.3: Formation of the performance vectors for each evaluated country**.

For each evaluated country i, the performance vector $\:{v}_{i}$ is generated by taking the worst-performing vector as a reference point:

12 $${v_i}={X_i} - {V^ - }=({x_{i1}} - v_{1}^{ - },{x_{i2}} - v_{2}^{ - }, \cdots ,{x_{in}} - v_{n}^{ - })$$

Here, $\:{X}_{i}=({x}_{i1},{x}_{i2},\dots\:,{x}_{in})$ represents the actual performance of country i.

**Step 5.4: Projection of performance vectors onto the base vector**.

The projection of $\:{v}_{i}$ onto $\:{v}^{*}$ gives $\:{E}_{i}$, indicating the country’s closeness to the ideal, is computed as follows:

13 $${E_i}=\frac{{v_{i}^{T}}}{{||{v^*}||}}=\frac{{\sum\nolimits_{{j=1}}^{n} {({x_{ij}} - v_{j}^{ - })(v_{j}^{+} - v_{j}^{ - })} }}{{\sqrt {\sum\nolimits_{{j=1}}^{n} {{{(v_{j}^{+} - v_{j}^{ - })}^2}} } }}$$

**Step 5.5: Calculation of the scale-invariant HDVP score**.

Finally, the HDVP score is normalized. It ranges between 0 and 1. This allows performance to be compared on a common scale.

14 $$HDVP{\text{ }}Score=\frac{{{E_i}}}{{||{v^*}||}}=\frac{{v_{i}^{T}{v^*}}}{{||{v^*}|{|^2}}}$$

A higher HDVP score means the performance is closer to the ideal, while a lower score indicates the opposite.

**Fig. 3:**
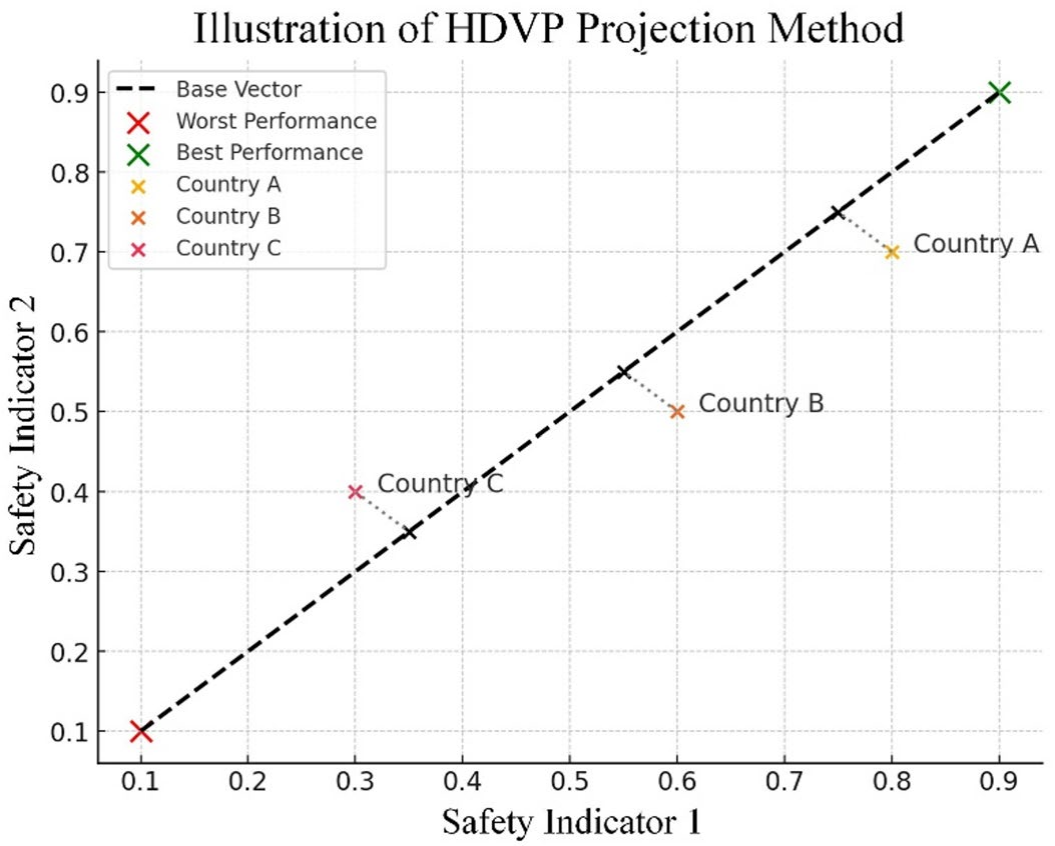
Geometry of HDVP.

Figure 3 shows the geometry of HDVP: each country’s performance vector is projected onto the line that connects the worst and best reference points.

**Step 6: Clustering using BeVarMax.**

Following Step 5, we have obtained a series of evaluation scores *E*_*i*_ (*i* = 1, 2,*…*,* m*) for all the *m* alternatives. On the basis of Ostu’s thresholding method^51^, we can treat all *E*_*i*_ values as a series of one-dimensional data, and then we could construct a histogram based on their values. Before building the histogram, we first obtain the maximum evaluation score as the upper-bound of intervals such that ${E^{\hbox{max} }}=\hbox{max} ({E_i})$ and the minimum evaluation score as the lower-bound of intervals such that ${E^{\hbox{min} }}=\hbox{min} ({E_i})$, and then divide $({E^{\hbox{max} }} - {E^{\hbox{min} }})$ into *L* consecutive small intervals where each interval has the same length equal to $\Delta E=\frac{{{E^{\hbox{max} }} - {E^{\hbox{min} }}}}{L}$ $\: \Delta E = \frac{{E^{{max}} - E^{{min}} }}{L}$ (typically set *L* = 50 in this case study). Thus, there are *L* intervals that are represented in ascending order as: $({E^{\hbox{min} }},{E^{\hbox{min} }}+\Delta E],({E^{\hbox{min} }}+\Delta E,{E^{\hbox{min} }}+2\Delta E], \cdots ,({E^{\hbox{max} }} - \Delta E,{E^{\hbox{max} }}]$.

Then, we let *N*_*l*_ (*l* = 1, 2, …, L) be the number of evaluation scores that lie within the *l*th interval, so obviously we have: $$\sum\limits_{{l=1}}^{L} {{N_l}} =m$$$$\: \sum\nolimits_{{l = 1}}^{L} {N_{l} = m}$$. Moreover, we can compute the probability of each interval by normalizing *N*_*l*_ by m such that: $${p_l}=\frac{{{N_l}}}{m}$$. In this study, assuming we have 3 clusters that are divided by the *k*_*1*_ -th and *k*_*2*_ -th intervals (for more clusters, the formulation would be similar and could be derived by analogy), the cumulative probability of the 3 clusters can be computed as:

15 $$\begin{gathered} {P_1}=\sum\limits_{{l=0}}^{{{k_1}}} {{p_l}} \hfill \\ {P_2}=\sum\limits_{{l={k_1}+1}}^{{{k_2}}} {{p_l}} \hfill \\ {P_3}=\sum\limits_{{l={k_2}+1}}^{{L - 1}} {{p_l}} \hfill \\ \end{gathered}$$

In addition, the cumulative mean of the 3 clusters could be given by:

16 $$\begin{gathered} {M_1}=\frac{1}{{{P_1}}}\sum\limits_{{l=0}}^{{{k_1}}} {l{p_l}} \hfill \\ {M_2}=\frac{1}{{{P_2}}}\sum\limits_{{l={k_1}+1}}^{{{k_2}}} {l{p_l}} \hfill \\ {M_3}=\frac{1}{{{P_3}}}\sum\limits_{{l={k_2}+1}}^{{L - 1}} {l{p_l}} \hfill \\ \end{gathered}$$

where we assume the 3 clusters are divided by the k1-th and k2-th intervals. The cumulative mean for all the data points can be written as: $M=\frac{1}{P}\sum\limits_{{i=0}}^{{L - 1}} {i{p_i}}$, $M = \frac{1}{P}\sum\nolimits_{{i = 0}}^{{L - 1}} {ip_{i} }$ where $P=\sum\limits_{{i=0}}^{{L - 1}} {{p_i}}$ $P = \sum\nolimits_{{i = 0}}^{{L - 1}} {p_{i} }$.

Then, by definition, the between-class variance can be defined as:

17 $${\sigma ^2}={P_1}{({M_1} - M)^2}+{P_2}{({M_2} - M)^2}+{P_3}{({M_3} - M)^2}$$

Note that the value of *σ* is controlled by the 2 variable interval indices *k*_1_ and *k*_2_ (as the cluster thresholds), so our problem is eventually equivalent to the following optimization problem:

18 $$\sigma 2(k_{1}^{*},k_{2}^{*})=\mathop {\hbox{max} }\limits_{{0<{k_1}<{k_2}<L - 1}} \sigma 2({k_1},{k_2})$$

This intuitively aims to seek the optimal interval index *k*_1_^∗^ and *k*_2_^∗^ that achieves the maximum between-class variance *σ*. In practical implementation, this could be solved by iterating all the possible *k*_1_ and *k*_2_ values to find the optimal ones that enable the maximum possible *σ*. Here, the computational complexity is *O*(*m*^2^), where *m* denotes the number of alternatives.

## Results and discussion

### Computational results

To comprehensively assess the road safety performance of the EAS countries, the proposed HDVP–BeVarMax model was applied to generate composite evaluation scores for each country across multiple years. These scores reflect the overall effectiveness of national safety initiatives and enable a robust comparison of performance trajectories over time. The following subsection presents the rankings and groups derived from these scores, offering insight into temporal progress and the relative positioning of countries within the EAS region.

#### Ranking

Based on the proposed HDVP–BeVarMax model, the overall performance scores of road safety for the 13 EAS countries are obtained, as shown in Table 2.

**Table 2:** Overall performance scores of the EAS countries and their respective rankings.

Country	ISO	2012		2015		2018		2023	
		Score	Rank	Score	Rank	Score	Rank	Score	Rank
**Brunei**	**BN**	0.0404	5	0.0462	4	0.0535	4	0.0395	5
**China**	**CN**	0.0201	9	0.0846	2	0.0352	6	0.0294	6
**Indonesia**	**ID**	0.0199	10	0.0107	10	0.0120	8	0.0112	9
**Japan**	**JP**	0.1138	1	0.1152	1	0.1412	1	0.1350	1
**Cambodia**	**KH**	0.0057	13	0.0083	13	0.0064	12	0.0041	12
**South Korea**	**KR**	0.0493	4	0.0458	5	0.0779	2	0.0679	3
**Laos**	**LA**	0.0108	12	0.0101	11	0.0084	11	0.0051	11
**Myanmar**	**MM**	0.0251	7	0.0092	12	0.0059	13	0.0032	13
**Malaysia**	**MY**	0.0293	6	0.0200	6	0.0266	7	0.0776	2
**Philippines**	**PH**	0.0769	2	0.0155	7	0.0484	5	0.0157	8
**Singapore**	**SG**	0.0506	3	0.0518	3	0.0620	3	0.0628	4
**Thailand**	**TH**	0.0169	11	0.0149	8	0.0115	9	0.0160	7
**Vietnam**	**VN**	0.0234	8	0.0120	9	0.0070	11	0.0068	10

As shown in Table 2, JP, KR, SG, and BN consistently achieved high rankings throughout the years, due to their long-established traffic safety systems, high compliance rates, strong enforcement infrastructure, and mature socio-economic conditions, which manifest their successful road safety performance. Conversely, MM, LA, TH, ID, and VN ranked relatively lower, which can be attributed to limited institutional capacity, underinvestment in road infrastructure, and challenges in law enforcement implementation, indicating that these countries may encounter more significant challenges and have a broader scope for improvements in road safety. Overall, these scores and rankings offer a comprehensive overview of road safety progress among the EAS countries over the specified period.

#### Grouping

The essence of measuring road safety success is rooted in the mutual learning between countries, especially for lower-performing nations to learn from those with the best performance. Thus, categorizing countries into distinct clusters that consist of countries with comparable advancements in terms of road safety could be very beneficial. By means of the proposed model, the 13 EAS countries are classified into three groups, as shown in Table 3.

**Table 3:** Groups of the EAS countries.

Country	2012	2015	2018	2023
**BN**	1	1	2	1
**CN**	2	2	2	2
**ID**	2	3	3	2
**JP**	1	1	1	1
**KH**	2	2	2	2
**KR**	2	2	2	2
**LA**	3	3	3	3
**MM**	3	3	3	3
**MY**	2	1	2	2
**PH**	2	3	2	3
**SG**	1	1	1	1
**TH**	3	3	3	3
**VN**	3	3	3	3

From Table 3, we can see that JP stably stays in the best-performing group, and KR and SG constantly stay in the medium group, whereas ID, KH, LA, MM, TH, and VN constantly stay in the worst-performing group in all 4 years.

### Robustness test

Robustness analyses involve testing the stability and reliability of the rankings and groupings obtained by comparing them with those derived from other analytical approaches. Stability refers to the extent to which the results obtained by the proposed model are disrupted by choosing different normalization techniques and weighting methods. Reliability refers to the consistency of the results obtained by the proposed model when compared with other benchmarking methods.

#### Comparison of ranking

##### Initial stability

To test the initial stability of the proposed model, we compare the rankings under different normalization methods: VE (Vector-based), MM (MinMax), and ZS (Z-Score), as shown in Table B. 1 (Appendix B).

As shown in Table B. 1, the ranking similarity across the three normalization methods is generally high, with strong consistency observed among top-ranked countries like Japan and Singapore, and bottom-ranked countries such as Cambodia, Myanmar, and Laos across all years. MM and ZS produce almost identical rankings, reflecting a strong alignment in how they scale and compare values. Overall, the ranking patterns are stable across methods, indicating the stability of the proposed model regardless of the normalization technique used.

**Fig. 4:**
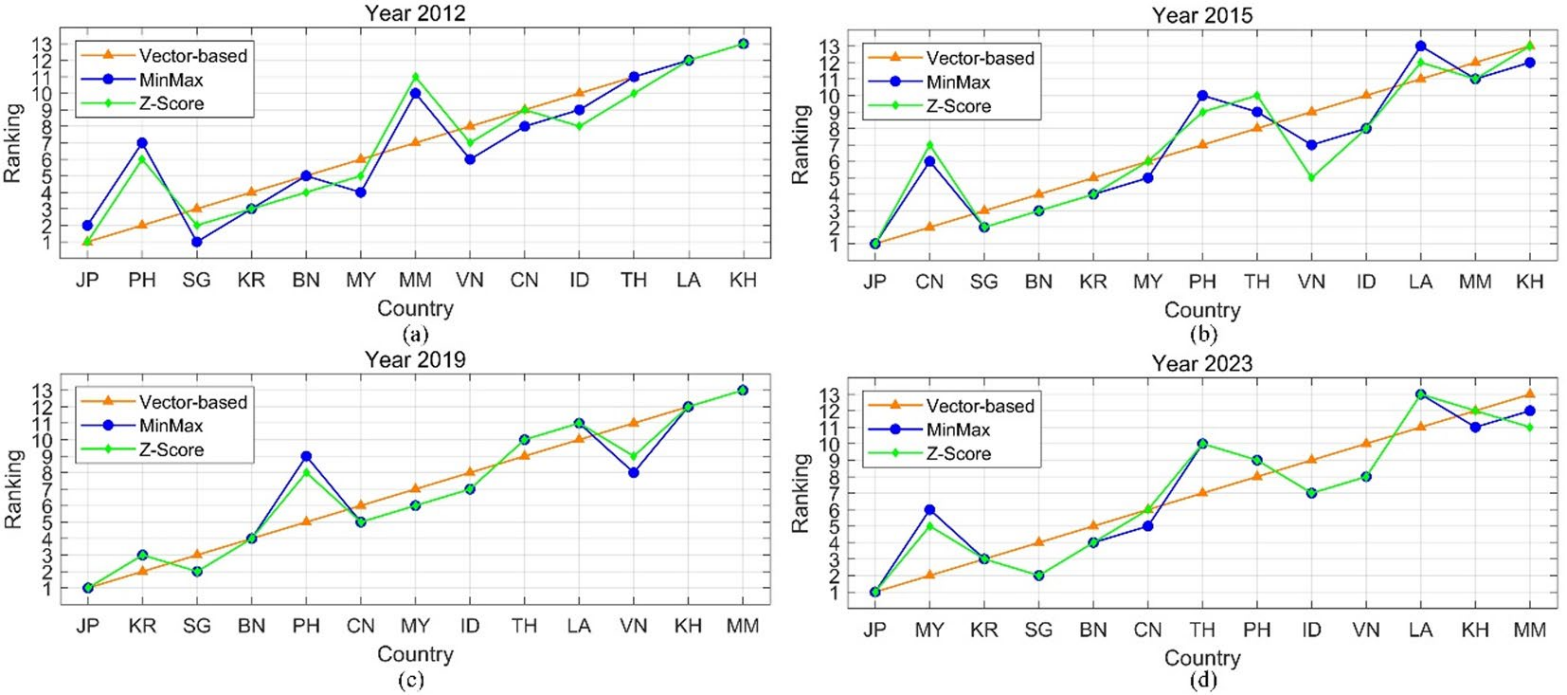
Rank contrast under different normalization techniques.

To more visually present the consistency of the rankings, line charts are introduced in Fig. 4.

As shown in Fig. 4, the ranking trajectories show a high degree of consistency, particularly for countries at the top (e.g., Japan) and bottom (e.g., Myanmar, Cambodia), which maintain stable positions across all methods and years. The VE method, represented by a smooth red line, generally produces a more linear and gradual ranking progression, whereas MM and ZS (green and blue lines, respectively) exhibit slightly more fluctuation, especially among mid-ranked countries such as China, the Philippines, and Thailand. Despite some divergence in individual years, particularly in 2015 and 2012, where ZS rankings display sharper variability, the overall trend across methods remains aligned, reflecting strong ranking similarity and robustness to the choice of normalization technique.

To more precisely assess the degree of similarity among the rankings generated by the various normalization techniques, Table 4 presents a correlation matrix.

**Table 4:** Correlation analyses of rankings obtained through various different normalization techniques.

Year	Method	MinMax	Z-Score	Vector
**2012**	**MinMax**	1		
	**Z-Score**	0.97	1	
	**Vector**	0.86	0.88	1
**2015**	**MinMax**	1		
	**Z-Score**	0.98	1	
	**Vector**	0.88	0.84	1
**2019**	**MinMax**	1		
	**Z-Score**	0.99	1	
	**Vector**	0.91	0.94	1
**2023**	**MinMax**	1		
	**Z-Score**	0.99	1	
	**Vector**	0.87	0.89	1

Table 4 illustrates a very high level of agreement among the rankings produced by the three normalization methods. In 2019, the correlation coefficients between MinMax and Z-Score, and between Vector and Z-Score, are 0.99 and 0.94, respectively, while the correlation between MinMax and Vector is slightly lower at 0.91. Similarly, in 2023, the correlations remain consistently high: 0.99 and 0.89 for both MinMax–Z-Score and Z-Score–Vector, and 0.87 for MinMax–Vector. These values, all close to 0.9, indicate an extremely strong positive relationship, suggesting that the rankings are highly consistent across the different normalization methods, with only minimal variation regardless of the year. This implies that the normalization technique has a negligible effect on the relative positioning of the evaluated entities based on the proposed model.

##### Intermediate stability

To test the intermediate stability of the proposed model, we compare the rankings under different weighting methods: DC (DCRITIC)^53^, EN (Entropy)^54^, and ME (MEREC)^55^, as shown in Table B. 2 (Appendix B).

As shown in Table B. 2, a high degree of similarity exhibits across the three weighting methods, DC, EN, and ME, over the years 2012, 2015, 2019, and 2023. Countries like Japan (JP) consistently maintain the top rank (1st) across all methods and years, reflecting strong robustness in evaluation regardless of the weighting approach. Similarly, nations such as Cambodia (KH), Laos (LA), and Myanmar (MM) remain at the lower end of the rankings with minimal fluctuation, indicating method-independent assessments for weaker performers. While most countries exhibit slight variations in their positions (typically within one or two ranks), the overall ranking structure is well-preserved across methods. Notable examples include South Korea (KR) and Singapore (SG), whose rankings show minor shifts but maintain a stable relative standing. This consistency implies that the choice of weighting method has a limited impact on the comparative ranking results, ensuring the stability of the proposed model.

**Fig. 5:**
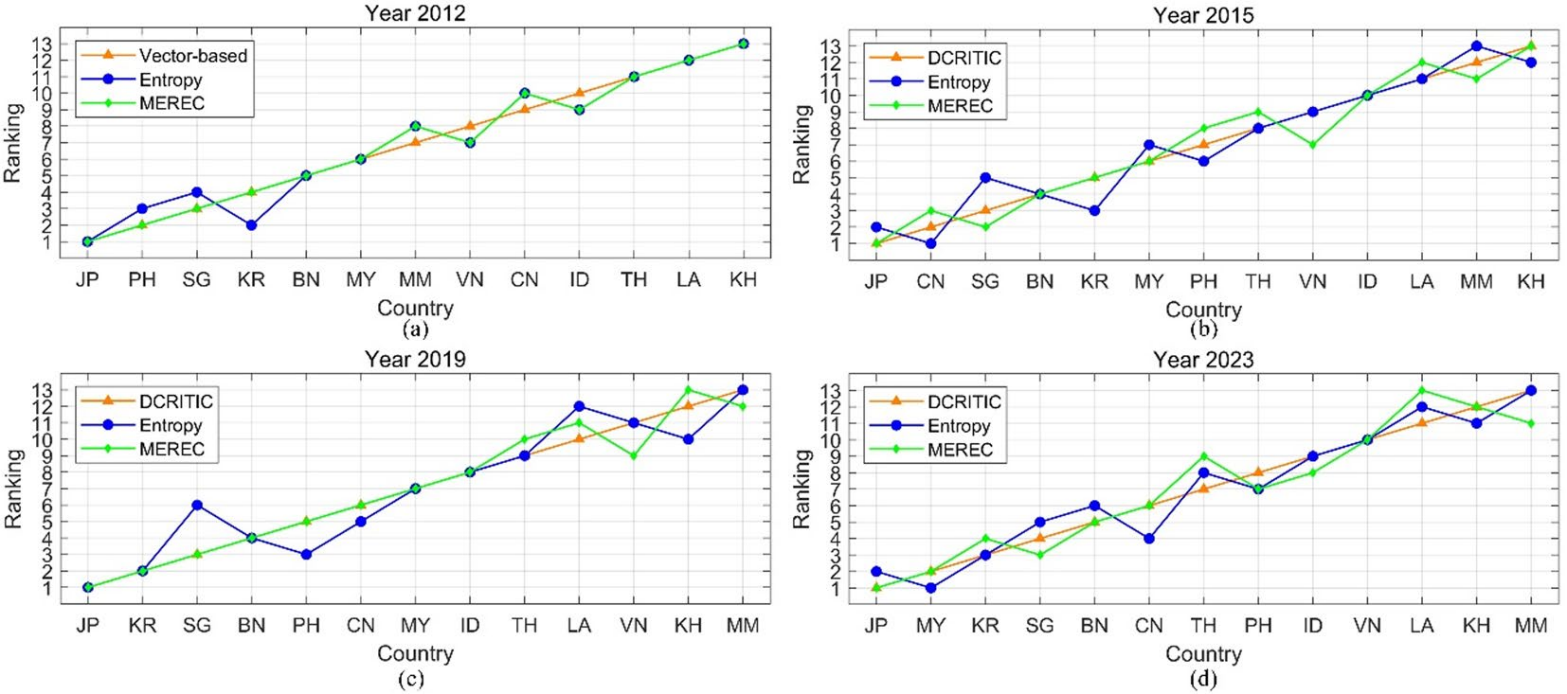
Rank contrast under different weighting techniques.

To more visually present the consistency of the rankings, line charts are introduced in Fig. 5.

As shown in Fig. 5, across all years, despite some fluctuations in the green (Entropy) and blue (MEREC) lines, particularly for mid-ranked countries like Singapore, the Philippines, and Vietnam, the overall ranking patterns are largely consistent across the three weighting methods. Notably, countries at the top (e.g., Japan) and bottom (e.g., Myanmar and Cambodia) maintain stable positions regardless of the weighting strategy, suggesting high agreement and robustness in the evaluation framework. The visual proximity of the curves implies a strong ranking similarity, with only slight deviations introduced by the specific characteristics of each weighting approach.

To more precisely assess the degree of similarity among the rankings generated by the various weighting techniques, Table 5 presents a correlation matrix.

**Table 5:** Correlation analyses of rankings obtained through various different weighting techniques.

Year	Method	DCRITIC	Entropy	MEREC
**2012**	**DCRITIC**	1		
	**Entropy**	0.98	1	
	**MEREC**	0.97	0.99	1
**2015**	**DCRITIC**	1		
	**Entropy**	0.91	1	
	**MEREC**	0.96	0.97	1
**2019**	**DCRITIC**	1		
	**Entropy**	0.92	1	
	**MEREC**	0.94	0.98	1
**2023**	**DCRITIC**	1		
	**Entropy**	0.95	1	
	**MEREC**	0.97	0.96	1

Table 5 illustrates a consistently high level of agreement among the rankings produced by the three weighting methods, DCRITIC, Entropy, and MEREC, across all four years (2012, 2015, 2019, and 2023). In 2012, correlations between methods range from 0.97 to 0.99, indicating near-identical ranking outputs. Although slightly lower in 2015, the correlations remain strong, with the lowest being 0.91 (DCRITIC vs. Entropy) and the highest at 0.97 (Entropy vs. MEREC). For both 2019 and 2023, the coefficients range from 0.92 to 0.98, reflecting a consistently high similarity in rankings. These results suggest that despite methodological differences in assigning weights, the resulting rankings are highly consistent, confirming the robustness and reliability of the evaluation regardless of the weighting approach employed.

##### Transverse reliability

To test the transverse reliability of the proposed model, we compare the rankings under the proposed model with two other benchmarking aggregating methods: TOPSIS^56^ and RSR^57^, as shown in Table B. 3 (Appendix B).

As shown in Table B. 3, the ranking similarity across these aggregating methods is quite strong, especially for countries with extreme performance. Japan (JP) consistently secures the top position across all methods and years, while Cambodia (KH), Laos (LA), and Myanmar (MM) frequently occupy the lower ranks, indicating a high level of robustness in their assessments. Minor variations appear in mid-ranked countries such as the Philippines (PH), Indonesia (ID), and Malaysia (MY), where rank positions differ slightly depending on the method used. For instance, PH fluctuates more under RSR compared to HDVP and TOPSIS. Despite these deviations, the general rank order among countries remains relatively stable, suggesting the reliability of the proposed model.

**Fig. 6:**
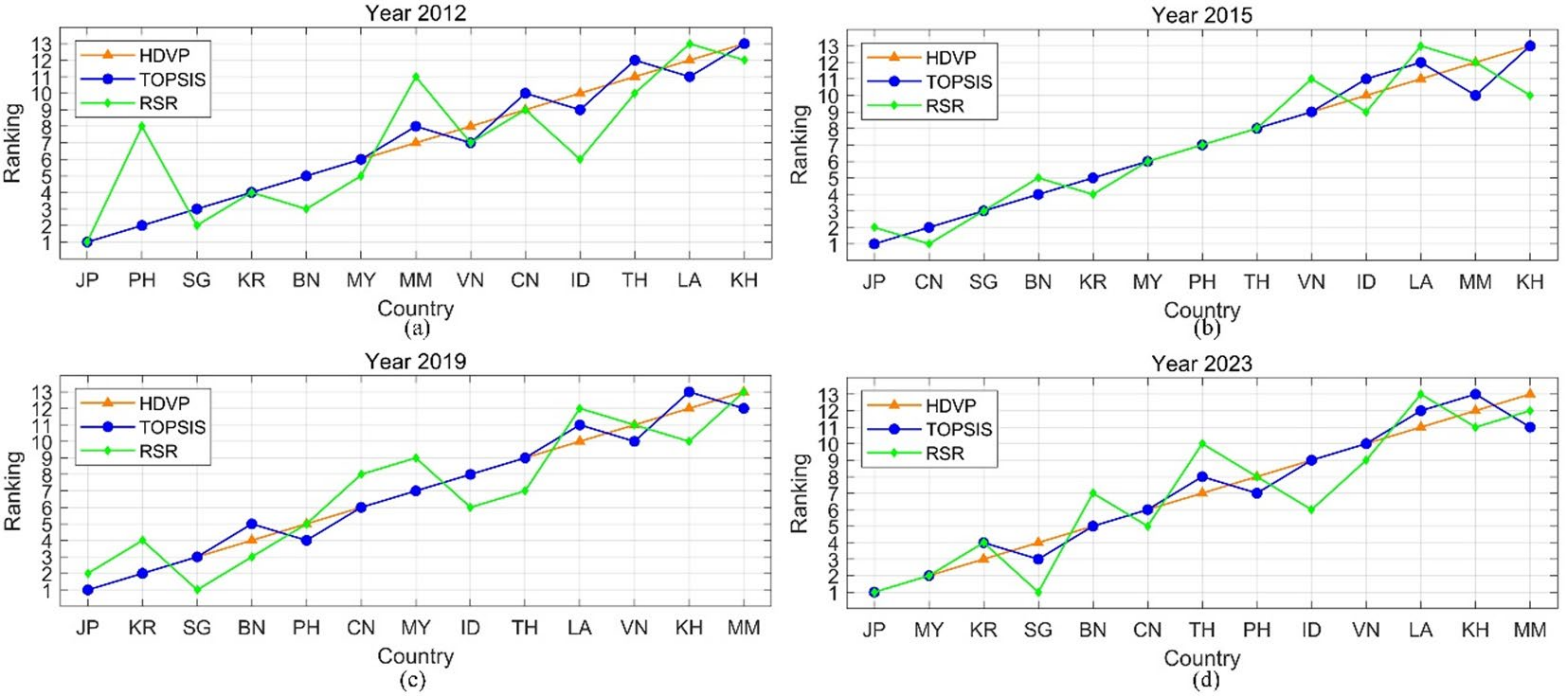
Rank contrast under different aggregating techniques.

To more visually present the consistency of the rankings, line charts are introduced in Fig. 6.

As shown in Fig. 6, HDVP generally aligns closely with TOPSIS (green line), although it occasionally diverges in the lower and middle ranks. Despite these variations, all three methods tend to agree on the top- and bottom-ranked countries. For example, Japan (JP) consistently ranks first, while Cambodia (KH) and Myanmar (MM) remain near the bottom across all methods and years. Overall, the aggregation methods produce largely similar rankings, indicating the reliability of the proposed model.

To more precisely assess the degree of similarity among the rankings generated by the various aggregating techniques, Table 6 presents a correlation matrix.

**Table 6:** Correlation analyses of rankings obtained through various different aggregating techniques.

Year	Method	HDVP	TOPSIS	RSR
**2012**	**HDVP**	1		
	**TOPSIS**	0.98	1	
	**RSR**	0.85	0.84	1
**2015**	**HDVP**	1		
	**TOPSIS**	0.98	1	
	**RSR**	0.81	0.79	1
**2019**	**HDVP**	1		
	**TOPSIS**	0.98	1	
	**RSR**	0.86	0.82	1
**2023**	**HDVP**	1		
	**TOPSIS**	0.97	1	
	**RSR**	0.83	0.87	1

Table 6 illustrates that a consistently high level of agreement among the rankings produced by the three aggregating techniques (HDVP, TOPSIS, and RSR) across the years 2012, 2015, 2019, and 2023. The correlation between HDVP and TOPSIS remains exceptionally strong throughout all years, consistently at 0.97 or 0.98, demonstrating nearly identical ranking outputs. While RSR shows slightly lower correlations with both HDVP and TOPSIS, its values still range from 0.79 to 0.87, indicating moderate to strong alignment. The correlation between RSR and the other methods is lowest in 2015, suggesting some divergence during that year. However, across all years, the coefficients are sufficiently high to confirm a substantial degree of ranking similarity, reinforcing the overall robustness and reliability of the proposed model.

#### Comparison of grouping

##### Initial stability

To test the initial stability of the proposed model in clustering, we compare the groups under different normalization methods: VE (Vector-based), MM (MinMax), and ZS (Z-Score), as shown in Table C. 1 (Appendix C).

As shown in Table C. 1, a high degree of group similarity exists among these methods. Countries such as Japan (JP) and Singapore (SG) are consistently assigned to Group 1 (green), regardless of normalization approach or year, reflecting strong consensus in identifying top performers. Conversely, Laos (LA) and Myanmar (MM) are uniformly placed in Group 3 (red), indicating robust agreement on their lower status. Most countries show minor shifts between Groups 1 and 2 or Groups 2 and 3, such as Indonesia (ID) and the Philippines (PH), which occasionally oscillate due to method-specific sensitivity. Nevertheless, the overall grouping structure remains stable, with only marginal differences across normalization techniques. This consistency underscores the stability of group classification outcomes based on the proposed model across different normalization schemes.

##### Transverse reliability

To test the transverse reliability of the proposed model, we compare the clusters under the proposed model with those from two benchmarking clustering methods: k-means and DBSCAN^58^, as shown in Table C. 2 (Appendix C).

As shown in Table C. 2, despite some method-specific variations, there is a generally high degree of group similarity among the clustering techniques. Countries such as Japan (JP) and Singapore (SG) are consistently assigned to Group 1 (green) across all methods and years, indicating strong consensus regarding their top performance. Similarly, Laos (LA), Myanmar (MM), Thailand (TH), and Vietnam (VN) are persistently classified into Group 3 (red), reflecting alignment in identifying lower-performing countries. Some moderate fluctuations are observed among mid-tier countries like China (CN), Indonesia (ID), and Malaysia (MY), where group assignments shift between Groups 1, 2, and 3 depending on the method. Overall, the consistency in group membership for both high and low performers demonstrates substantial similarity across clustering approaches, reinforcing the reliability of the classification based on the proposed model.

## Policy and practical guidance

### De-composition of composite score changes

**Fig. 7:**
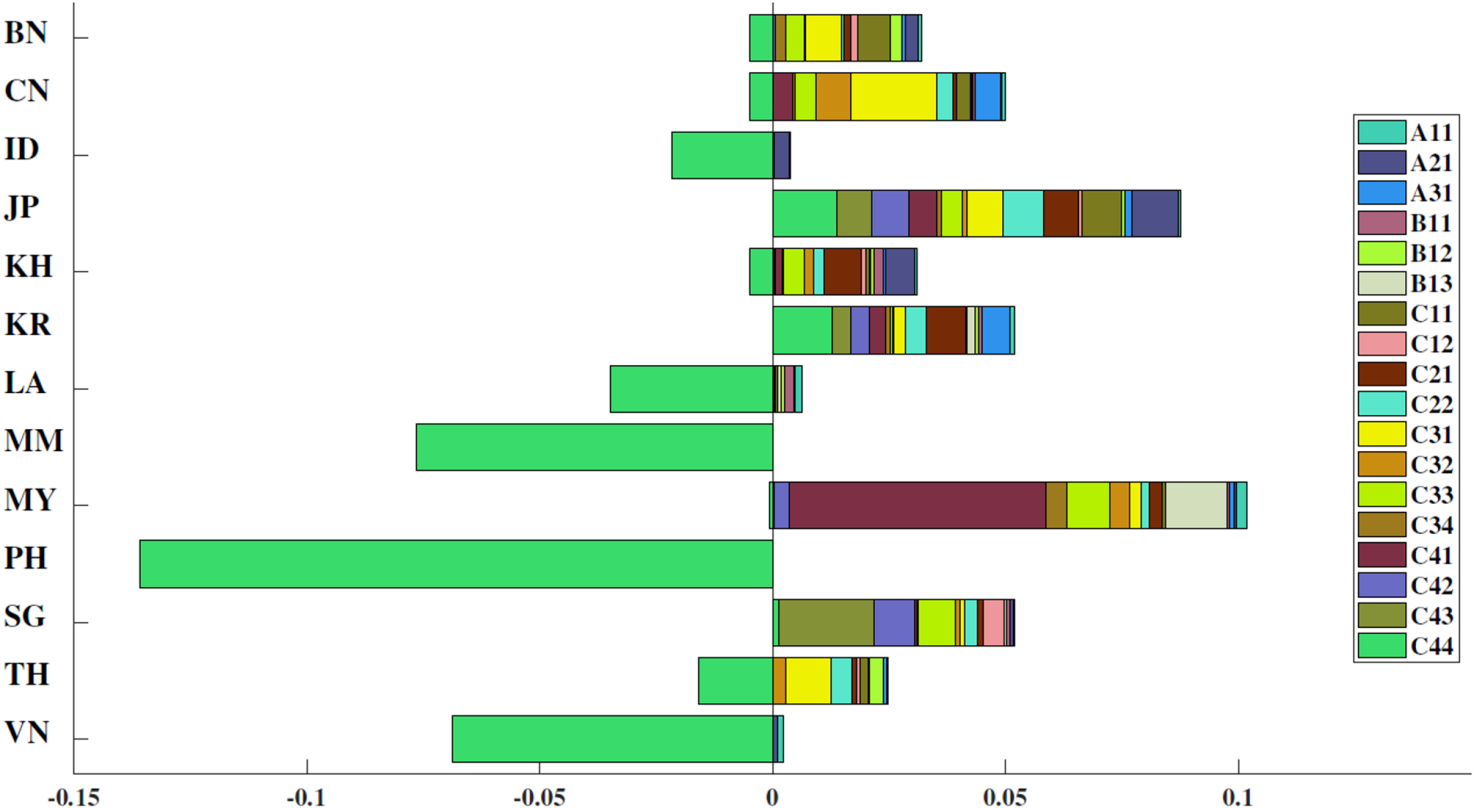
De-composition of composite score changes for each country.

De-composition of score changes involves breaking down the total change in a score over time (or between scenarios) into individual contributing factors or components. This analytical approach helps identify why a score changed, how much each factor contributed to the change, and what areas drove performance improvement or decline, as presented in Fig. 7.

As shown in Fig. 7, countries such as PH, MM, and VN show pronounced negative changes, largely driven by high traffic fatality rates (A11, A21), weak law enforcement (C41–C44), and low vehicle standards (C12). Conversely, countries such as SG, JP, and KR exhibit strong positive performance, with contributing factors including high seatbelt and helmet use (B12, B13), robust socio-economic indicators (C32–C34), and comprehensive traffic law enforcement. For example, JP’s sustained top performance is closely tied to its long-standing investments in traffic safety culture, high public compliance, and a mature legal system with rigorous enforcement. MY stands out with notable improvements due to enhancements in socio-economic metrics (e.g., GDP per capita and literacy rates), but its performance is moderated by lagging infrastructure indicators (C21, C22). The significant variability reflects each nation’s governance capacity, urbanization level, and policy implementation. Emerging economies (e.g., LA, KH) often suffer from institutional and infrastructural gaps, limiting their ability to enforce laws and upgrade vehicle standards. In contrast, developed countries leverage stronger institutional frameworks, public awareness, and technological integration to improve road safety outcomes.

Figure 7 also predominantly showcases an overall improvement in road safety for the majority of regions analyzed. The positive extensions signify not just incremental advancements but possibly reflect the success of sustained, long-term road safety initiatives such as improved vehicle safety standards, enhanced driver training programs, infrastructure upgrades, and rigorous law enforcement. This trend underscores the effectiveness of concerted efforts in elevating road safety as a public health priority.

The variation in the composition of these bars across different countries suggests that certain interventions have been more effective or prioritized in some respects over others. By showing insightful references to the challenges that require attention and the necessary actions, this de-composition process provides policymakers with a roadmap for enhancing road safety performance in their respective countries.

### Benchmarking within each cluster

**Fig. 8:**
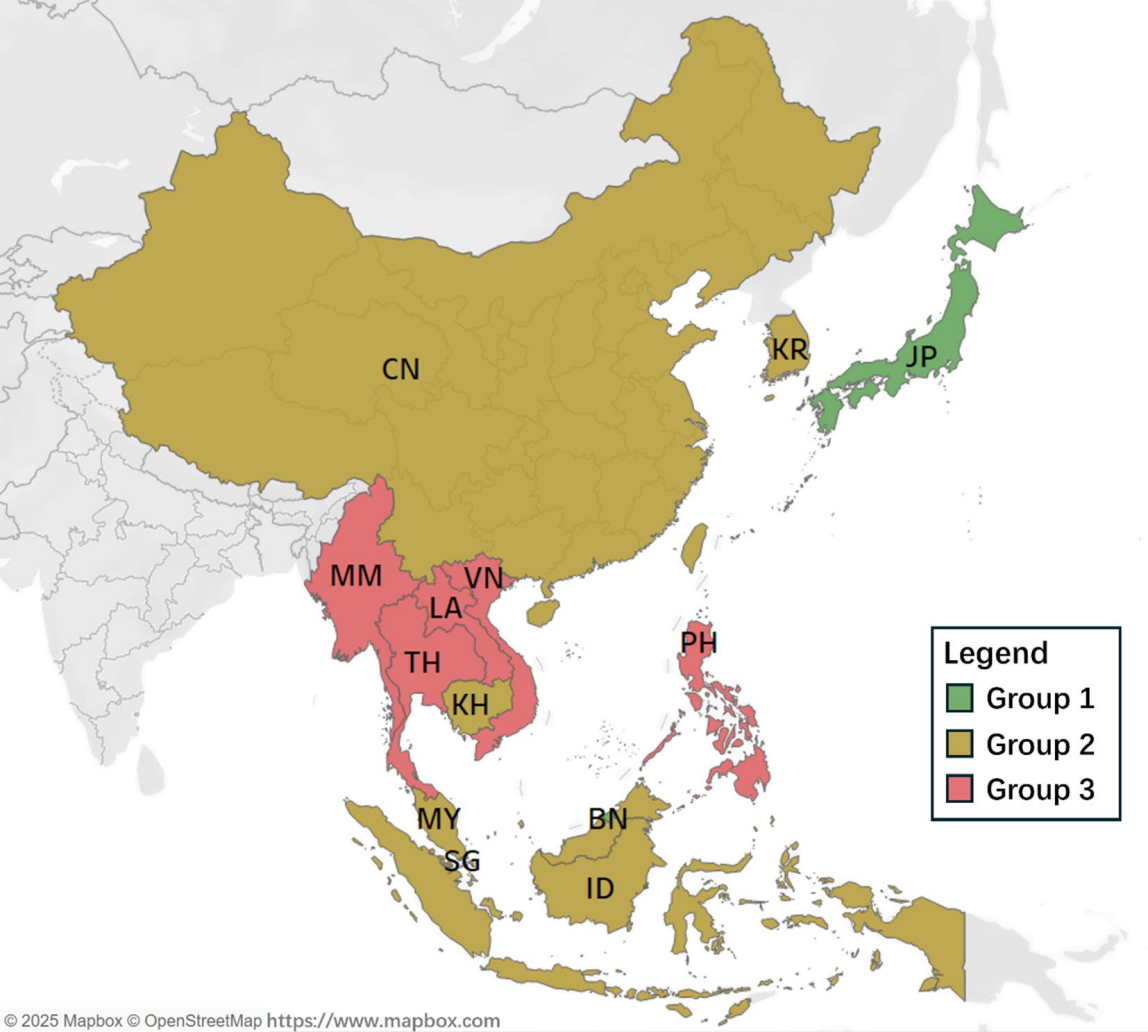
Groups distribution in geography.

**Fig. 9:**
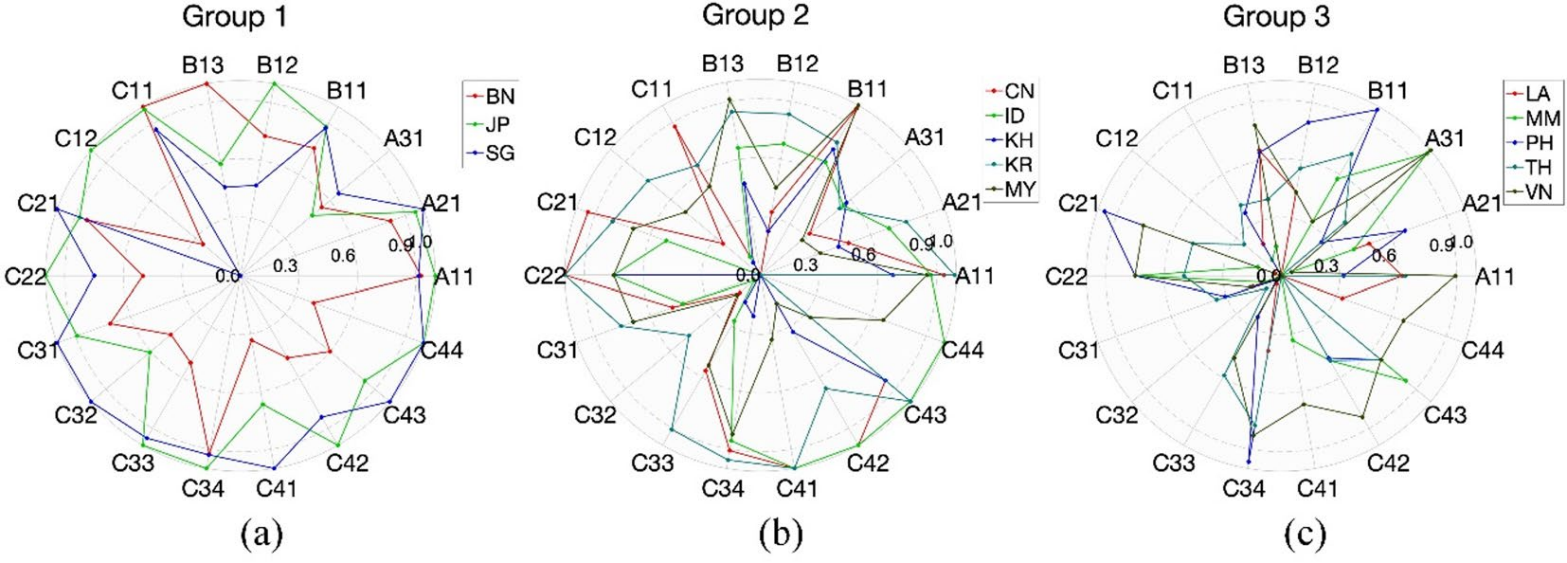
Benchmarking of SPIs within each cluster.

To enhance the ability of less successful nations to adopt effective road safety practices from the top performers, it is essential to dissect the EAS countries’ overall performance into specific indicators. For this purpose, each group received a radar chart that clearly displays their performance on various SPIs relative to other nations within the same cluster. This method simplifies the learning process, as the countries within a group have broadly similar overall achievements. The detailed breakdown of individual road safety performance indicators for the EAS countries by group is illustrated in Figs. 8 and 9.

As shown in Figs. 8 and 9:

In Group 1 (Fig. 8(a)), Japan (JP) and Singapore (SG) generally exhibit higher and more consistent scores across most indicators, especially in behavioral (B11–B13) and socio-economic (C31–C34) dimensions. Brunei (BN) demonstrates strong performance in several indicators (e.g., B13, C11) but shows notable weaknesses in areas such as vehicles and enforcement (C12, C22) and road infrastructure (C21). This visual comparison allows for the identification of relative strengths and deficiencies among the countries, providing insights for targeted improvements within the cluster. They should also pilot cutting-edge safety innovations, such as AI-based enforcement, connected vehicle technologies, and smart infrastructure, and rigorously evaluate their outcomes to serve as scalable models. Moreover, enhancing the granularity of data collection (e.g., disaggregated by demographic and geography) can help fine-tune domestic policy and offer refined templates for transferability to other contexts.

In Group 2 (Fig. 8(b)), South Korea (KR) and China (CN) generally show stronger performance across enforcement (C22), behavioral (B11–B13), and socio-economic indicators (C33–C34), while Cambodia (KH) displays more variability, with relatively lower scores in several areas such as road infrastructure (C21, C22) and socio-economic dimensions (C31–C32). Malaysia (MY) performs well in some behavioral and enforcement indicators but less consistently across others. Indonesia (ID) shows moderate performance overall, with a few peaks in infrastructure and regulatory components. To enhance consistency and elevate group-level performance, this group should focus on harmonizing policy implementation across subnational regions. Specific actions include strengthening cross-sector coordination (e.g., transport, health, education), expanding road audits (C22) in rapidly urbanizing areas, and investing in community-based behavior change interventions. Countries like Malaysia can further enhance their influence by acting as regional mentors in enforcement design, while Cambodia may benefit from external technical support and donor-backed investment in road and vehicle safety infrastructure.

In Group 3 (Fig. 8(c)), the Philippines (PH) shows relatively stronger outcomes in behavioral indicators (B11–B13) and road infrastructure (C21), while Vietnam (VN) scores comparatively higher in several regulatory and enforcement areas (e.g., C41–C44). Thailand (TH) and Myanmar (MM) display more moderate and fluctuating performances, with scattered strengths across various dimensions. In contrast, Laos (LA) shows relatively lower values across most indicators, highlighting key areas needing improvement. Within this group, there is a spread of performance across different indicators, with no single country consistently leading or lagging in all areas. This suggests that while these countries face challenges in road safety, there are specific areas where each country performs relatively well and can be a source of best practices for others. Immediate priorities include enacting and uniformly enforcing foundational road safety laws (C41–C44), upgrading critical rural and peri-urban road networks (C21), and adopting minimum vehicle safety standards (C11–C12). Governments should integrate road safety goals into broader development programs, leveraging health, education, and transport sectors to achieve multiplier effects. Regional cooperation is key; these countries should seek targeted capacity-building partnerships with Group 1 and 2 nations and establish annual monitoring systems to ensure sustained improvement and accountability.

Overall, the radar chart effectively captures the multidimensional performance profiles of countries (i.e., strengths, weaknesses, and variability in safety performance) within each cluster and provides insights into areas for targeted policy development, capacity building, and resource allocation. This kind of benchmarking encourages a collaborative approach to improving road safety, where countries learn from each other’s successes and implement strategies that have been effective elsewhere.

### Policy lessons for low-performing countries

For low-performing countries such as Myanmar (MM), Laos (LA), Vietnam (VN), and Cambodia (KH), the de-composition analysis underscores several critical areas requiring strategic attention and targeted intervention. These countries consistently rank in the lowest cluster group across all years, with significant negative contributions from high traffic fatality rates (A11, A21), weak enforcement of safety laws (C41–C44), low vehicle standards (C12), and underdeveloped road infrastructure (C21, C22). Drawing on the benchmarking insights and performance structures of higher-performing peers within the same region, several policy lessons can be distilled.

First, strengthening institutional capacity for traffic law enforcement is paramount. Countries like Japan, Singapore, and South Korea exhibit strong enforcement performance across all four legal dimensions, i.e., national speed limits, drink-driving, seatbelt, and helmet laws, which strongly correlate with their high road safety scores. Low performers should prioritize legislative reforms, ensure legal clarity, and invest in consistent, data-driven enforcement strategies to increase compliance and deterrence.

Second, improving behavioral compliance and public safety awareness must be addressed. Enhancing helmet and seatbelt usage (B12, B13) through nationwide awareness campaigns, school-based education programs, and media outreach can produce tangible safety improvements. For example, Vietnam’s moderate scores in enforcement contrast with relatively better outcomes in helmet use, suggesting that behavioral change is feasible even in resource-constrained settings.

Third, infrastructure investments must align with safety objectives. Low percentages of paved roads (C21) and a lack of systematic safety audits (C22) hinder the ability to create a safe travel environment. Countries should channel infrastructure budgets toward expanding and upgrading critical road networks, integrating safety audits, and deploying low-cost, high-impact improvements such as signage, lighting, and pedestrian facilities.

Fourth, socio-economic development is a foundational enabler of long-term road safety gains. Higher income levels, literacy rates, and life expectancy (C31–C34) often correlate with stronger road safety systems. While structural economic reforms are long-term goals, integrating road safety into broader development programs, such as health, education, and rural access, can yield synergistic effects.

Lastly, regional cooperation and peer learning offer cost-effective strategies for capacity building. Mechanisms for sharing best practices, harmonizing standards, and conducting joint training workshops, particularly within EAS frameworks, can help low-performing countries emulate successful interventions from peers in similar socio-political contexts.

In summary, tailored strategies that combine legislative reform, behavior change, infrastructure development, and cross-country collaboration hold the key to accelerating safety improvements in underperforming countries.

## Concluding remarks

### Conclusion

This study proposes a scientific HDVP–BeVarMax model as an effective MCDM framework to measure road safety performance and aid policy-making and strategic planning with stability and reliability. Utilizing a practical case focused on road safety management within the EAS region, multiple empirical comparisons underscore the strength and reliability of the developed model, validating its effectiveness, relevance, and versatility in real-world MCDM applications. Through the prioritization and classification of nations based on their aggregate road safety scores, the model highlights those that have shown marked improvement throughout the past ten years. The de-composition of performance reveals the intricate contributions of diverse attributes to each country’s road safety score, offering insights into areas where funding and strategic initiatives should be directed for maximum impact. Moreover, benchmarking (i.e., the identification of best practices) according to each indicator further empowers policymakers with knowledge of effective measures, promoting evidence-based policy planning and implementation. Overall, this model not only facilitates composite evaluation at the regional level but also sets a benchmark for road safety performance looking into the future. It equips policymakers, managers, and decision-makers with a robust framework for decision-making and strategic planning.

This study offers significant contributions to both academic inquiry and public sector management. The development of a brand-new framework for measuring road safety performance incorporates aggregation, classification, de-composition, and benchmarking mechanisms, thereby enhancing the evaluation process and enriching the MCDM mechanisms. It provides methodological support for EAS countries in risk factor identification, detection of underlying problems, and prioritization of interventions. The empirical findings derived from this approach equip decision-makers and policymakers in the EAS countries with the necessary insights to strengthen their strategic planning and policy implementation efforts. This, in turn, promotes greater political commitment and reinforces cross-sectoral accountability, ultimately supporting the formulation of impactful policies and the execution of effective interventions aimed at improving road safety outcomes.

### Limitations and future studies

Despite the methodological advancements and comprehensive scope of the proposed HDVP–BeVarMax framework, several limitations should be acknowledged. First, the model relies primarily on available quantitative data, which may not fully capture qualitative aspects such as governance quality or cultural factors influencing road safety outcomes. Second, the model’s applicability may be constrained in contexts where data are sparse or inconsistent across countries or time periods. Third, although we demonstrate the model’s reliability through comparative analysis, a more rigorous sensitivity analysis, as outlined in recent studies (e.g^59,60^. would provide deeper insights into how weighting influences the model’s outcomes. Incorporating such an analysis could help identify the most influential parameters and further enhance confidence in the model’s generalizability.

Future research could extend the present framework in several meaningful directions. First, incorporating qualitative variables, such as institutional capacity, stakeholder engagement, or enforcement culture, would provide a more holistic understanding of road safety performance. The integration of fuzzy logic or probabilistic modeling could also enhance the model’s ability to handle uncertainty, particularly in contexts with incomplete or imprecise data. Second, expanding the analysis to other regions or conducting cross-regional comparisons could test the model’s robustness and adaptability across diverse socio-economic environments. Third, future studies might explore the dynamic effects of specific policy interventions over time by integrating time-series or panel-data methodologies. Additionally, enhancing the model’s participatory dimension by involving policymakers and local experts in the weighting processes could improve both the relevance and practical uptake of the proposed decision-support framework.

## Supplementary Information

Below is the link to the electronic supplementary material.

Supplementary Material 1

## Data Availability

Data and code will be made available upon reasonable request from the corresponding author.
