# Supplementary material for: Road safety measurement with reliability using an advanced hybrid decision model
Source: Sci Rep. 2025 Oct 8;15:35099. doi: 10.1038/s41598-025-18918-7 (PMC12508111; doi:10.1038/s41598-025-18918-7)
Supplement: Supplementary file 1 — Supplementary Material 1 [file 41598_2025_18918_MOESM1_ESM.docx]

# Appendix A

Table A. 1 provides classical methods used for aggregating MCDM operations. Table A. 2 provides a summary of the frequently used grouping strategies.

Table A. 1 Classical aggregating methods.

| **Year** | **Method** | **Abbreviation** |
| --- | --- | --- |
| 1763 | Taxonomy | Taxonomy |
| 1922 | Weighted Product Model | WPM |
| 1959 | Condorcet-Kemeny-Young-Levenglick ranking procedure | CKYL |
| 1971 | Analytic Hierarchy Process | AHP |
| 1971 | DEcision MAking Trial and Evaluation Laboratory | DEMATEL |
| 1975 | QUALIFLEX | QUALIFLEX |
| 1976 | Multi-Attribute Utility Theory | MAUT |
| 1978 | Data Envelopment Analysis | DEA |
| 1979 | Alternative Queuing Method | AQM |
| 1980 | Organization, Rangement et synthèse dedonnées relarionnelles | ORESTE |
| 1980s | Osculating Value Method | OVM |
| 1981 | Technique for Order of Preference by Similarity to Ideal Solution | TOPSIS |
| 1982 | EVAluation of MIXed data | EVAMIX |
| 1982 | Grey Relation Analysis | GRA |
| 1983 | REGIME | REGIME |
| 1986 | Preference Ranking Organization METHod for Enrichment Evaluation | PROMETHEE |
| 1988 | Rank-Sum Ratio | RSR |
| 1989 | Concordance Analysis | CA |
| 1990 | ELimination and Et Choice Translating REality | ELECTRE |
| 1990 | measuring attractiveness through a categorical-based evaluation technique | MACBETH |
| 1991 | EXtension of the PROMethee | EXPROM |
| 1992 | interactive and multi-criteria decision making | TODIM |
| 1994 | COmplex PRoportional Assessment | COPRAS |
| 1994 | Evidential Reasoning Approach | ERA |
| 1995 | Conflict Analysis Method | CAM |
| 1996 | Analytic Network Process | ANP |
| 1996 | PAMSSEM | PAMSSEM |
| 1998 | Non-Structural Fuzzy Decision Support System | NSFDSS |
| 1998 | vise kriterijumska optimizacija i kompromisno resenje | VIKOR |
| 2001 | Superiority and Inferiority Ranking | SIR |
| 2002 | Principal component analysis of scores Relative to an Identified Distribution Integral Transformation | PRIDIT |
| 2004 | Multi-Objective Optimization on the basis of Ratio Analysis | MOORA |
| 2010 | Additive Ratio Assessment | ARAS |
| 2010 | Stepwise Weight Assessment Ratio Analysis | SWARA |
| 2012 | Weighted Aggregated Sum Product ASsessment | WASPAS |
| 2014 | KEmeny Median Indicator Ranks Accordance | KEMIRA |
| 2015 | Best-Worst Method | BWM |
| 2015 | Evaluation based on Distance from Average Solution | EDAS |
| 2015 | Multi-Attributive Border Approximation area Comparison | MABAC |
| 2016 | COmbinative Distance based ASsessment | CODAS |
| 2018 | COmbined COmpromise Solution | COCOSO |
| 2020 | Measurement of Alternatives and Ranking according to COmpromise Solution | MARCOS |
| 2020 | Order Rated Effectiveness | ORE |
| 2022 | Dombi Bonferroni | DOBI |
| 2022 | Multiplicative Multi-Objective Ratio Analysis | MULTIMOORA |
| 2023 | Alternative Ranking Order Method Accounting for two-Step Normalization | AROMAN |
| 2023 | Compromise Ranking of Alternatives from Distance to Ideal Solution | CRADIS |

Table A. 2 Classical grouping methods.

| **Year** | **Method** | **Abbreviation** |
| --- | --- | --- |
| — | Joint Singular value de-composition and Semi-discrete de-composition | JSS |
| 1873 | Singular Value De-composition | SVD |
| 1894 | Gaussian Mixture Model | GMM |
| 1901 | Principal Component Analysis | PCA |
| 1932 | Clustering Analysis | CA |
| 1936 | Linear Discriminant Analysis | LDA |
| 1951 | K-Nearest Neighbors | KNN |
| 1960s | Multiple Correspondence Analysis | MCA |
| 1973 | Fuzzy C-Means | FCM |
| 1975 | Mean Shift Clustering | MSC |
| 1983 | Semi-Discrete De-composition | SDD |
| 1988 | Rank-Sum Ratio | RSR |
| 1994 | Non-Negative Matrix Factorization | NNMF |
| 2002 | Principal component analysis of scores Relative to an Identified Distribution Integral Transformation | PRIDIT |
| 2007 | Affinity Propagation Clustering | APC |

# Appendix B

Table B. 1 Ranking comparisons using different normalization methods across years.

| **Country** | **2012** |  |  | **2015** |  |  | **2019** |  |  | **2023** |  |  |
| --- | --- | --- | --- | --- | --- | --- | --- | --- | --- | --- | --- | --- |
|  | **VE** | **MM** | **ZS** | **VE** | **MM** | **ZS** | **VE** | **MM** | **ZS** | **VE** | **MM** | **ZS** |
| **BN** | 5 | 5 | 4 | 4 | 3 | 3 | 4 | 4 | 4 | 5 | 4 | 4 |
| **CN** | 9 | 8 | 9 | 2 | 6 | 7 | 6 | 5 | 5 | 6 | 5 | 6 |
| **ID** | 10 | 9 | 8 | 10 | 8 | 8 | 8 | 7 | 7 | 9 | 7 | 7 |
| **JP** | 1 | 2 | 1 | 1 | 1 | 1 | 1 | 1 | 1 | 1 | 1 | 1 |
| **KH** | 13 | 13 | 13 | 13 | 12 | 13 | 12 | 12 | 12 | 12 | 11 | 12 |
| **KR** | 4 | 3 | 3 | 5 | 4 | 4 | 2 | 3 | 3 | 3 | 3 | 3 |
| **LA** | 12 | 12 | 12 | 11 | 13 | 12 | 10 | 11 | 11 | 11 | 13 | 13 |
| **MM** | 7 | 10 | 11 | 12 | 11 | 11 | 13 | 13 | 13 | 13 | 12 | 11 |
| **MY** | 6 | 4 | 5 | 6 | 5 | 6 | 7 | 6 | 6 | 2 | 6 | 5 |
| **PH** | 2 | 7 | 6 | 7 | 10 | 9 | 5 | 9 | 8 | 8 | 9 | 9 |
| **SG** | 3 | 1 | 2 | 3 | 2 | 2 | 3 | 2 | 2 | 4 | 2 | 2 |
| **TH** | 11 | 11 | 10 | 8 | 9 | 10 | 9 | 10 | 10 | 7 | 10 | 10 |
| **VN** | 8 | 6 | 7 | 9 | 7 | 5 | 11 | 8 | 9 | 10 | 8 | 8 |
| *Note*: VE=Vector-based, MM=MinMax, and ZS=Z-Score. | | | | | | | | | | | | |

Table B. 2 Ranking comparisons using different weighting methods across years.

| **Country** | **2012** |  |  | **2015** |  |  | **2019** |  |  | **2023** |  |  |
| --- | --- | --- | --- | --- | --- | --- | --- | --- | --- | --- | --- | --- |
|  | **DC** | **EN** | **ME** | **DC** | **EN** | **ME** | **DC** | **EN** | **ME** | **DC** | **EN** | **ME** |
| **BN** | 5 | 5 | 5 | 4 | 4 | 4 | 4 | 4 | 4 | 5 | 6 | 5 |
| **CN** | 9 | 10 | 10 | 2 | 1 | 3 | 6 | 5 | 6 | 6 | 4 | 6 |
| **ID** | 10 | 9 | 9 | 10 | 10 | 10 | 8 | 8 | 8 | 9 | 9 | 8 |
| **JP** | 1 | 1 | 1 | 1 | 2 | 1 | 1 | 1 | 1 | 1 | 2 | 1 |
| **KH** | 13 | 13 | 13 | 13 | 12 | 13 | 12 | 10 | 13 | 12 | 11 | 12 |
| **KR** | 4 | 2 | 4 | 5 | 3 | 5 | 2 | 2 | 2 | 3 | 3 | 4 |
| **LA** | 12 | 12 | 12 | 11 | 11 | 12 | 10 | 12 | 11 | 11 | 12 | 13 |
| **MM** | 7 | 8 | 8 | 12 | 13 | 11 | 13 | 13 | 12 | 13 | 13 | 11 |
| **MY** | 6 | 6 | 6 | 6 | 7 | 6 | 7 | 7 | 7 | 2 | 1 | 2 |
| **PH** | 2 | 3 | 2 | 7 | 6 | 8 | 5 | 3 | 5 | 8 | 7 | 7 |
| **SG** | 3 | 4 | 3 | 3 | 5 | 2 | 3 | 6 | 3 | 4 | 5 | 3 |
| **TH** | 11 | 11 | 11 | 8 | 8 | 9 | 9 | 9 | 10 | 7 | 8 | 9 |
| **VN** | 8 | 7 | 7 | 9 | 9 | 7 | 11 | 11 | 9 | 10 | 10 | 10 |
| *Note*: DC=DCRITIC, EN=Entropy, and ME=MEREC. | | | | | | | | | | | | |

Table B. 3 Ranking comparisons using different aggregating methods across years.

| **Country** | | **2012** |  |  | **2015** |  |  | **2019** |  |  | **2023** |  |  |
| --- | --- | --- | --- | --- | --- | --- | --- | --- | --- | --- | --- | --- | --- |
|  |  | **HDVP** | **TOPSIS** | **RSR** | **HDVP** | **TOPSIS** | **RSR** | **HDVP** | **TOPSIS** | **RSR** | **HDVP** | **TOPSIS** | **RSR** |
| **BN** | 5 | 5 | 3 | 4 | 4 | 5 | 4 | 5 | 3 | 5 | 5 | 7 |  |
| **CN** | 9 | 10 | 9 | 2 | 2 | 1 | 6 | 6 | 8 | 6 | 6 | 5 |  |
| **ID** | 10 | 9 | 6 | 10 | 11 | 9 | 8 | 8 | 6 | 9 | 9 | 6 |  |
| **JP** | 1 | 1 | 1 | 1 | 1 | 2 | 1 | 1 | 2 | 1 | 1 | 1 |  |
| **KH** | 13 | 13 | 12 | 13 | 13 | 10 | 12 | 13 | 10 | 12 | 13 | 11 |  |
| **KR** | 4 | 4 | 4 | 5 | 5 | 4 | 2 | 2 | 4 | 3 | 4 | 4 |  |
| **LA** | 12 | 11 | 13 | 11 | 12 | 13 | 10 | 11 | 12 | 11 | 12 | 13 |  |
| **MM** | 7 | 8 | 11 | 12 | 10 | 12 | 13 | 12 | 13 | 13 | 11 | 12 |  |
| **MY** | 6 | 6 | 5 | 6 | 6 | 6 | 7 | 7 | 9 | 2 | 2 | 2 |  |
| **PH** | 2 | 2 | 8 | 7 | 7 | 7 | 5 | 4 | 5 | 8 | 7 | 8 |  |
| **SG** | 3 | 3 | 2 | 3 | 3 | 3 | 3 | 3 | 1 | 4 | 3 | 1 |  |
| **TH** | 11 | 12 | 10 | 8 | 8 | 8 | 9 | 9 | 7 | 7 | 8 | 10 |  |
| **VN** | 8 | 7 | 7 | 9 | 9 | 11 | 11 | 10 | 11 | 10 | 10 | 9 |  |

# Appendix C

Table C. 1 Clustering comparisons using different clustering methods across years.

| **Country** | **2012** |  |  | **2015** |  |  | **2019** |  |  | **2023** |  |  |
| --- | --- | --- | --- | --- | --- | --- | --- | --- | --- | --- | --- | --- |
|  | **VE** | **MM** | **ZS** | **VE** | **MM** | **ZS** | **VE** | **MM** | **ZS** | **VE** | **MM** | **ZS** |
| **BN** | 1 | 1 | 1 | 1 | 1 | 1 | 2 | 1 | 1 | 1 | 1 | 1 |
| **CN** | 2 | 2 | 2 | 2 | 2 | 1 | 2 | 2 | 2 | 2 | 2 | 2 |
| **ID** | 2 | 2 | 2 | 3 | 2 | 1 | 3 | 2 | 2 | 2 | 2 | 2 |
| **JP** | 1 | 1 | 1 | 1 | 1 | 1 | 1 | 1 | 1 | 1 | 1 | 1 |
| **KH** | 2 | 2 | 3 | 2 | 2 | 2 | 2 | 2 | 2 | 2 | 2 | 2 |
| **KR** | 2 | 2 | 2 | 2 | 2 | 2 | 2 | 2 | 2 | 2 | 1 | 2 |
| **LA** | 3 | 3 | 3 | 3 | 3 | 3 | 3 | 3 | 3 | 3 | 3 | 3 |
| **MM** | 3 | 3 | 3 | 3 | 3 | 3 | 3 | 3 | 3 | 3 | 3 | 3 |
| **MY** | 2 | 1 | 1 | 1 | 1 | 1 | 2 | 1 | 1 | 2 | 1 | 1 |
| **PH** | 2 | 2 | 2 | 3 | 2 | 2 | 2 | 2 | 2 | 3 | 2 | 2 |
| **SG** | 1 | 1 | 1 | 1 | 1 | 1 | 1 | 2 | 1 | 2 | 1 | 1 |
| **TH** | 3 | 2 | 2 | 3 | 3 | 2 | 3 | 3 | 3 | 3 | 3 | 3 |
| **VN** | 3 | 3 | 3 | 3 | 3 | 3 | 3 | 3 | 3 | 3 | 3 | 3 |
| *Note*: VE=Vector-based, MM=MinMax, and ZS=Z-Score. | | | | | | | | | | | | |

Table C. 2 Clustering comparisons using different clustering methods across years.

| **Country** | **2012** |  |  | **2015** |  |  | **2019** |  |  | **2023** |  |  |
| --- | --- | --- | --- | --- | --- | --- | --- | --- | --- | --- | --- | --- |
|  | **BeVarMax** | **K-means** | **DBSCAN** | **BeVarMax** | **K-means** | **DBSCAN** | **BeVarMax** | **K-means** | **DBSCAN** | **BeVarMax** | **K-means** | **DBSCAN** |
| **BN** | 1 | 1 | 1 | 1 | 2 | 1 | 2 | 1 | 1 | 1 | 2 | 1 |
| **CN** | 2 | 2 | 2 | 2 | 2 | 1 | 2 | 2 | 2 | 2 | 2 | 2 |
| **ID** | 2 | 3 | 2 | 3 | 2 | 2 | 3 | 2 | 2 | 2 | 2 | 2 |
| **JP** | 1 | 1 | 1 | 1 | 1 | 1 | 1 | 1 | 1 | 1 | 1 | 1 |
| **KH** | 2 | 2 | 2 | 2 | 2 | 2 | 2 | 2 | 2 | 2 | 2 | 2 |
| **KR** | 2 | 2 | 2 | 2 | 1 | 2 | 2 | 1 | 2 | 2 | 1 | 1 |
| **LA** | 3 | 3 | 3 | 3 | 3 | 3 | 3 | 3 | 3 | 3 | 3 | 3 |
| **MM** | 3 | 3 | 3 | 3 | 3 | 3 | 3 | 3 | 3 | 3 | 3 | 3 |
| **MY** | 2 | 1 | 2 | 1 | 1 | 1 | 2 | 1 | 1 | 2 | 1 | 2 |
| **PH** | 2 | 3 | 2 | 3 | 3 | 2 | 2 | 3 | 2 | 3 | 2 | 3 |
| **SG** | 1 | 1 | 1 | 1 | 1 | 1 | 1 | 1 | 1 | 1 | 1 | 1 |
| **TH** | 3 | 3 | 3 | 3 | 3 | 2 | 3 | 3 | 3 | 3 | 3 | 3 |
| **VN** | 3 | 3 | 3 | 3 | 3 | 3 | 3 | 3 | 3 | 3 | 3 | 3 |
